# RNA demethylase ALKBH5 prevents pancreatic cancer progression by posttranscriptional activation of PER1 in an m6A-YTHDF2-dependent manner

**DOI:** 10.1186/s12943-020-01158-w

**Published:** 2020-05-19

**Authors:** Xingya Guo, Kai Li, Weiliang Jiang, Yangyang Hu, Wenqin Xiao, Yinshi Huang, Yun Feng, Qin Pan, Rong Wan

**Affiliations:** 1 Department of Gastroenterology, Shanghai General Hospital, Shanghai Jiaotong University School of Medicine, No. 100, Haining Road, Shanghai, 200080 People’s Republic of China; 2 Department of Gastroenterology, Xinhua Hospital, Shanghai Jiaotong University School of Medicine, No. 1665, Kongjiang Road, Shanghai, 200092 People’s Republic of China

**Keywords:** Pancreatic cancer, m6A, ALKBH5, PER1

## Abstract

**Background:**

N6-methyladenosine (m6A) is the most abundant reversible methylation modification of eukaryotic mRNA, and it plays vital roles in tumourigenesis. This study aimed to explore the role of the m6A demethylase ALKBH5 in pancreatic cancer (PC).

**Methods:**

The expression of ALKBH5 and its clinicopathological impact were evaluated in PC cohorts. The effects of ALKBH5 on the biological characteristics of PC cells were investigated on the basis of gain-of-function and loss-of-function analyses. Subcutaneous and orthotopic models further uncovered the role of ALKBH5 in tumour growth. mRNA and m6A sequencing and assays of m6A methylated RNA immunoprecipitation-qPCR (MeRIP-qPCR) were performed to identify the targeted effect of ALKBH5 on PER1. P53-binding sites in the ALKBH5 promoter were investigated by ChIP and luciferase assays to reveal the interplay between ALKBH5 and PER1-activated ATM-CHK2-P53/CDC25C signalling.

**Results:**

ALKBH5 loss characterized the occurrence and poor clinicopathological manifestations in patients with PC. Overexpression of ALKBH5 reduced tumoural proliferative, migrative, invasive activities in vitro and ameliorated tumour growth in vivo, whereas ALKBH5 knockdown facilitated PC progression. Mechanistically, ALKBH5 posttranscriptionally activated PER1 by m6A demethylation in an m6A-YTHDF2-dependent manner. PER1 upregulation led to the reactivation of ATM-CHK2-P53/CDC25C signalling, which inhibited cell growth. P53-induced activation of ALKBH5 transcription acted as a feedback loop regulating the m6A modifications in PC.

**Conclusion:**

ALKBH5 serves as a PC suppressor by regulating the posttranscriptional activation of PER1 through m6A abolishment, which may highlight a demethylation-based approach for PC diagnosis and therapy.

## Background

Pancreatic cancer (PC), a highly malignant gastrointestinal tumour characterized by insidious onset, difficulty in early diagnosis and low surgical resection rate, has become one of the most common lethal tumours worldwide [1–3]. The 5-year survival rate of PC is only 8%, and even descents to 3% at the distant metastasis stage [4]. Therefore, it is of urgent need to illuminate the molecular and cellular mechanisms underlying PC with the purpose of diagnostic and therapeutic intervention.

Currently, it is well known that epigenetic modification plays an important role in the initiation and progression of tumours. *N*6-methyladenosine (m6A), first reported in the 1970s [5, 6], is the most prevalent modification of the epitranscriptome in eukaryotic cells [7]. m6A modifications reflect a dynamic and reversible process [8, 9] that is introduced by the m6A methyltransferase complex (MTC) composing methyltransferase-like 3 (METTL3), METTL14 and Wilms tumour 1-associated protein (WTAP), and is demethylated by human AlkB homolog H5 (ALKBH5) and fat mass and obesity-associated protein (FTO) [8–12]. Accumulating proofs confirm that the m6A modification regulates multiple biological functions, such as RNA processing, nuclear export, translation, degradation and RNA-protein interactions [9, 13–15], whereas aberrant m6A modification underlies embryonic development disorders, tumourigenesis, failures in homeostasis and differentiation of immune cells, and nervous system diseases [16–19]. Moreover, self-renewal of stem cells, proliferative promotion, and chemoresistance dominate the m6A-related actions in various cancers [20–22]. In addition, m6A modification functions in condition of its recognition by m6A reader proteins (YT521-B homology (YTH) domain family [15], heterogeneous nuclear ribonucleoprotein (hnRNP)) [23].

Serving as the key demethylase of m6A modification, ALKBH5 acts to promote the tumour progression by maintaining the stemness of breast cancer cells [20]. Contrastively, shallow/deep deletion of ALKBH5 correlates with cytogenetic abnormalities, P53 mutation, and lowered overall survival (OS) and event-free survival (EFS) in acute leukaemia [24]. These effects highlight an important, yet contradictory, impact of ALKBH5 on malignant diseases. However, the characteristics of ALKBH5-dependent m6A modification and its pathological role in PC remain to be illustrated.

In the present study, we demonstrated the expressive loss of ALKBH5 with correlation to deteriorated clinicopathological factors and survival outcome in PC patients. Decreased ALKBH5 resulted in tumour growth and invasion by the inhibition of PER1-ATM-CHK2-P53/CDC25C signalling in an m6A-YTHDF2-dependent manner. Then, inactivation of the ALKBH5-PER1-P53-ALKBH5 feedback loop reflected another aspect of ALKBH5-related disorder of m6A methylation in PC progression.

## Methods

### Cell culture

Five human PC cell lines (Aspc-1, Bxpc-3, SW1990, CFPAC and Panc-1) commercially obtained from American Type Culture Collection (Rockville, MD, USA) were cultured in high-glucose Dulbecco’s modified Eagle’s medium (HyClone, Logan, UT, USA) containing 10% foetal bovine serum (Gibco, Grand Island, NY, USA) in a 5% CO_2_ environment at 37 °C. HPDE6c7, an immortalized human pancreatic duct epithelial cell line, was offered by Kyushu University, Japan and cultured by keratinocyte serum-free medium supplemented with an epidermal growth factor and bovine pituitary extract (Gibco). All these cell lines were routinely authenticated for purity and being infection-free.

### Patients and specimens

A total of 42 PC tissues and adjacent normal tissues were collected from PC patients undergoing standard resection, without chemotherapy or radiotherapy, at Shanghai General Hospital from 2010 to 2014 with 5-year follow-up (Table 1). Clinicopathological parameters (e.g., tumour differentiation, TNM stage, vascular invasion, and ALKBH5 expression) were then subjected to univariate and multivariate Cox regression analysis in purpose of uncovering the independent risk factors for overall survival time. The study was approved by the Ethics Committee of Shanghai General Hospital and carried out in accordance with the Declaration of Helsinki Principles. All patients provided written informed consent before enrollment.

**Table 1:** Correlation between ALKBH5 expression and clinicopathological features of pancreatic cancer (PC)

Characteristic	No. of patients (%)	ALKBH5 expression		*P
		Low ***n*** = 22 (%)	High ***n*** = 20(%)	
Age				
<60	20 (47.6)	12 (54.5)	8 (40.0)	0.346
≥60	22 (52.4)	10 (45.5)	12 (60.0)	
Gender				
Male	19 (45.2)	10 (45.5)	9 (45.0)	0.976
Female	23 (54.8)	12 (54.5)	11 (55.0)	
Tumor location				
Head	29 (69.0)	15 (68.2)	14 (70.0)	0.899
Body/tail	13 (31.0)	7 (31.8)	6 (30.0)	
Tumor size				
≥3cm	14 (33.3)	9 (40.9)	5 (25.0)	0.275
<3cm	28 (66.7)	13 (59.1)	15 (75.0)	
Differentiation				
Well to moderate	28 (66.7)	11 (50.0)	17 (85.0)	0.016*
Poor	14 (33.3)	11 (50.0)	3 (15.0)	
^a^TNM stage				
I	11 (26.2)	3 (13.6)	8 (40.0)	0.033*
II	17 (40.5)	8 (36.4)	9 (45.0)	
III	14 (33.3)	11 (50.0)	3 (15.0)	
Vascular invasion				
Absent	24 (57.1)	9 (40.9)	15 (75.0)	0.026*
Present	18 (42.9)	13 (59.1)	5 (25.0)	

^a^There is no patients with TNM IV stage tumors

*P<0.05 indicates a significant relationship among the variables

### Vector construction and transduction

The full-length of PER1 sequence was cloned into a PmirGLO dual luciferase expression vector (Promega, Madison, WI, USA) containing Renilla luciferase (R-luc) and firefly luciferase (F-luc) to construct a wild-type PER1 reporter plasmid. To build the PER1 mutant reporter plasmid, adenosine bases within the m6A consensus sites were replaced by cytosine, and the amplified ALKBH5 promoter region with wild-type or mutated P53-binding sites was subcloned into a pGL3 basic vector (Promega). Second, the pGMLV-PA6 vector (Genomeditech, Shanghai, China) was employed to construct the ALKBH5-expressing lentivirus (Lv-ALKBH5). shALKBH5 containing ALKBH5-targeting shRNA was constructed by pGMLV-SC5RNAi lentiviral vector (Genomeditech). The target sequence of ALKBH5 was 5′-GAAGCTTCAATGGTCTCCTTA-3′. A scrambled shRNA targeting 5′-TTCTCCGAACGTGTCACGT-3′ was used as a negative control. Stably transfected cells were selected with puromycin (Sigma-Aldrich, St Louis, MO, USA). In addition, lentiviral vectors expressing human PER1 (Lv-PER1), P53 (Lv-P53), YTHDF2-specific shRNA (shYTHDF2), empty vectors (vector), and plasmids containing scrambled shRNA (scramble) were constructed as previously described [25–27].

### RNA sequencing

mRNA sequencing was applied to ALKBH5-expressing (Lv-ALKBH5) and control BxPC-3 cells (Vector) using HiSeq-2000 with single-end reads with read lengths of 50 bp (PE50) (Illumina Inc., San Diego, CA, USA). Sequencing data were then subjected to FPKM value assessment (fragments per kilobase mRNA sequence per million mapped reads) of each gene by TopHat-Cufflinks (v2.2.1) [28]. Analysis of gene ontology (GO) and signalling pathways were subsequently performed using DAVID (www.david.niaid.nih.gov) and KEGG databases (http://www.genome.jp/kegg/), respectively.

### M6A-RNA immunoprecipitation (MeRIP) assay and m6A sequencing

Total RNA was extracted from BxPC-3 cells with ALKBH5 (BxPC-3/Lv-ALKBH5) or empty vector control (BxPC-3/Vector) overexpression, and treated with DNase (Sigma) to remove genomic DNA. After mRNA purification and fragmentation, the fragments were incubated with m6A primary antibody for immunoprecipitation using a Magna MeRIP™ m6A kit (#17–10,499, Merck Millipore, MA, USA). Enriched m6A modified mRNA was then detected either through qRT-PCR or by next generation sequencing using Illumina HiSeq 2000 (Illumina Inc.). Subsequently, raw sequence data were trimmed and filtered by Trim Galore! (http://www.bioinformatics.babraham.ac.uk/projects/trim_galore/) with default parameters. Following quality check by FastQC v0.11.3 (http://www.bioinformatics.bbsrc.ac.uk/projects/fastqc), the TopHat-Cufflinks (v2.2.1) program mapped reads to the human reference genome (GRCh37/hg19). The proposed peak calling MACS algorithm (version 1.4.0rc2) with parameters, −format = “SAM” --gsize = 2.82e9 --tsize = 36 --nomodel --shiftsize = 100 -to-large False -w –S was used to find m6A peaks.

### m6A quantification

Total m6A mRNA levels are colourimetrically measured by ELISA assay with an EpiQuik m6A RNA Methylation Quantification kit (Epigentek, Farmingdale, NY, USA). Then, 200 ng of purified polyA+ mRNA was added for the analysis of each sample. Measurements were performed in triplicate following the manufacturer’s instructions.

### Luciferase reporter assay

PC cells were transfected with a luciferase reporter, the pRL-TK Renilla luciferase construct (Promega), and the ALKBH5 or P53 expression vectors. The ratios of firefly and Renilla luciferase activities were determined 48 h post-transfection using a Dual-Luciferase Assay kit (Promega).

### Quantitative real-time RT-PCR

Total RNA was isolated from clinical specimens or human PC cells using Trizol reagent (Invitrogen, Carlsbad, CA, USA), and then RNA was used to perform reverse transcription with a Transcriptor First Strand cDNA Synthesis kit (Roche, Basel, Switzerland). The acquired cDNAs were used as templates for quantitative real-time PCR analysis using SYBR Green PCR Master Mix (Qiagen, Valencia, CA, USA). The relative RNA expression levels were calculated using the 2-ΔΔCt method, with the levels normalized to GAPDH mRNA. The specific primers used in this study are listed in supplementary Table 1.

### Western blotting

Cells were harvested and dissolved in RIPA lysis buffer, and the protein concentrations were determined using a bicinchoninic acid (BCA) protein assay (Beyotime Biotechnology, Jiangsu, China). Whole cell lysates were fractionated and transferred to PVDF membranes by an electroblot apparatus. Membranes were incubated at 4 °C overnight with specific primary antibodies. Bands were visualized with an ECL detection reagent (Beyotime). The densitometric quantification was analysed with a β-actin control using Image Lab software (Bio-Rad, Hercules, CA, USA). All antibodies used in this study were obtained from Santa Cruz (CA, USA) and Abcam (Cambridge, UK). The primary antibodies were rabbit monoclonal anti-ALKBH5 (ab195377, Abcam), mouse monoclonal anti-PER1 (sc-398,890, Santa Cruz), mouse monoclonal anti-p-ATM (Ser-1981; sc-47,739, Santa Cruz), rabbit monoclonal anti-p-CHK2 (Thr-68; ab32148, Abcam), rabbit polyclonal anti-p-CDC25C (Ser216; ab47322, Abcam), rabbit monoclonal anti-p-P53 (Ser-15; ab1431, Abcam), mouse monoclonal anti-P21 (sc-71,811, Santa Cruz), mouse monoclonal anti-CYCLIN B1 (ab72, Abcam), rabbit polyclonal anti-p-CDK1 (Tyr15; ab47594), mouse monoclonal anti-CDK1 (A17, Abcam) and rabbit polyclonal anti-β-ACTIN (ab8227, Abcam).

### In vitro cell proliferation, migration and invasion assays

First, a cell counting kit-8 (CCK-8; Dojindo, Kumamoto, Japan) and a 5-ethynyl-20-deoxyuridine assay (EdU) kit (Cell Light EdU DNA imaging Kit, RiboBio, Guangzhou, China) were used to evaluate the proliferative activity of PC cells according to the manufacturer’s instructions. Second, colony formation assays were conducted to evaluate the long-term proliferation of PC cells as described in our previous study [29]. Third, wound-healing assays were carried out by generating a vertical scratch on a monolayer of PC cells. Images were captured under an inverted microscope after 24 h, and the wound area was calculated in five randomly selected microscopic fields. Additionally, transwell chamber assays were performed to assess cell invasive ability in accordance with the method described in our previous study [29].

### Flow cytometric analysis

PC cells were collected 48 h after transfection. Successively, they were fixed in ice-cold 70% ethanol overnight, stained with 50 mg/ml propidium iodide (BD Biosciences, San Jose, CA, USA) in the dark for 30 min at 4 °C and finally analysed by flow cytometry (FACSCalibur, BD Biosciences, San Jose, CA, USA) and Modfit software (Verity Software House, Topsham, ME, USA).

### Subcutaneous and orthotopic implantation of PC model

A cohort of 20 male nude mice was randomly assigned to groups of SW1990/shALKBH5, SW1990/scramble, BxPC-3/Lv-ALKBH5 and BxPC-3/vector (*n* = 5 per group), respectively. Equal numbers of corresponding cells (2 × 10^6^ per mouse) were injected subcutaneously in the right flank to establish a PC xenograft model. Tumour volumes were monitored twice per week after the end of the first week. Mice were finally sacrificed at week 4 to harvest the tumour bulks. In addition, ALKBH5-knockdown SW1990 cells, ALKBH5-overexpressing BxPC-3 cells, and the corresponding wild-type cells were subjected to luciferase labelling. A total of 3 × 10^6^ cells were suspended in a 50 μl volume with a mixture of serum-free medium and high concentration Matrigel (v/v, 1:1) and were orthotopically injected into the tail of the pancreas in each mouse from the different groups. In vivo tumour growth was monitored with a Xenogen IVIS Illumina System (Caliper Life Sciences, Hopkinton, MA, USA) for 4 weeks.

### Co-Immunoprecipitation (CoIP) assay

293 T cells transfected with PER1 mRNA or an empty vector were treated with immunoprecipitation (IP) lysis buffer on ice for 30 min and then centrifuged at 12,000 rpm for 15 min at 4 °C. After an aliquot subjected to protein expression analyses, the supernatant was precleared for 1 h at 4 °C with protein A-sepharose beads (GE Healthcare, Chicago, IL, USA). IP was performed with the addition of an ATM antibody (sc-377,293, Santa Cruz) or with control IgG and protein A-sepharose beads; the mixes were incubated overnight at 4 °C with gentle rotation. Beads were then washed with lysis buffer three times, and the supernatants were harvested for western blotting.

### Chromatin immunoprecipitation (ChIP) assay

SW1990 and BxPC-3 cells infected with P53 expression vectors or the negative controls were cross-linked using 1% formaldehyde and then were lysed in SDS lysis buffer. The sonicated cell lysates were then mixed with chip dilution buffer and precleared with protein A-agarose/salmon sperm DNA (Millipore) for 30 min. The recovered supernatant was incubated with either an anti-P53 monoclonal antibody (ab1101, Abcam) or an isotype control IgG overnight at 4 °C. Next, immunoprecipitated complexes were precipitated and washed. Cross-linking of immunoprecipitates was reversed at 65 °C for 4 h, which was followed by treatment with RNase A and 100 μg/mL proteinase K at 50 °C for 3 h for DNA fragment recycling. Extracted DNA samples were finally dissolved in TE buffer and subjected to PCR analysis.

### Immunohistochemistry and immunofluorescence

Human PC and xenograft tumour tissue paraffin-embedded sections were deparaffinized and rehydrated in succession, and then they were incubated with monoclonal antibodies against ALKBH5 (1:200, ab195377, Abcam), MMP-2 (1:250, ab86607, Abcam), MMP-9 (1:250, ab76003, Abcam) or Ki-67 (1:200, sc-23,900, Santa Cruz), respectively, at 4 °C overnight. Thereafter, biotinylated secondary antibodies were added for 30 min. For double immunofluorescence experiments, sections or cells were incubated with specific antibodies against ALKBH5 (1:200, ab195377, Abcam) and PER1 (1:100, sc-398,890, Santa Cruz), which was followed by incubation with the indicated fluorophore-conjugated secondary antibody. For m6A staining, the primary antibody m6A (1:2000, 202,111, Synaptic Systems) was first used, and then there was incubation with the secondary antibody (goat anti-rabbit IgG (H + L). Nuclear counterstaining was conducted with DAPI, and digital images were obtained using a fluorescence microscope (Leica, Germany).

ALKBH5 and PER1 signals in immunohistochemical staining were evaluated by a semi-quantitative scoring system based on the product of positive percentage and signal intensity [30]. The positive percentage was defined as follow: 0 = 0%, 1 = 1–25%, 2 = 26–50%, 3 = 51–75%, 4 = above 75%. The signal intensity was defined as follow: 0 = no staining, 1 = weak staining, 2 = intermediate staining, 3 = strong staining. Then, the median value of ALKBH5 and PER1 scoring in all specimens was chosen as the cut-off value. Specimen with ALKBH5 and/or PER1 score lower than the cut-off value was classified as the low-expression one; otherwise, it was classified as the high-expression one. The quantification of IHC staining was performed by 2 trained pathologists who were not aware of the experiments.

### Statistical analysis

Statistical analyses were performed by SPSS version 22 software (SPSS Inc., Chicago, IL, USA) for clinical analyses and GraphPad Prism 7.0 (GraphPad Software, La Jolla, CA, USA) for experimental analyses. The results are shown as the means ± SD of at least three biological replicates. Comparisons between two groups were analysed by Student’s t-tests. One-way analysis of variance (ANOVA) followed by Dunnett’s test was used for comparisons among multiple groups. The relationship between ALKBH5 and PER1 expression levels was determined using Pearson correlation analysis. OS curves were plotted to estimate survival based on the Kaplan-Meier method, and then a log-rank test was adopted for comparison. The relationship between ALKBH5 levels and clinicopathological factors was determined using chi-squared and Fisher’s exact test. A *P*-value less than 0.05 was considered to be statistically significant for all tests.

## Results

### ALKBH5 loss characterized PC with predictive and prognostic values

**Fig. 1:**
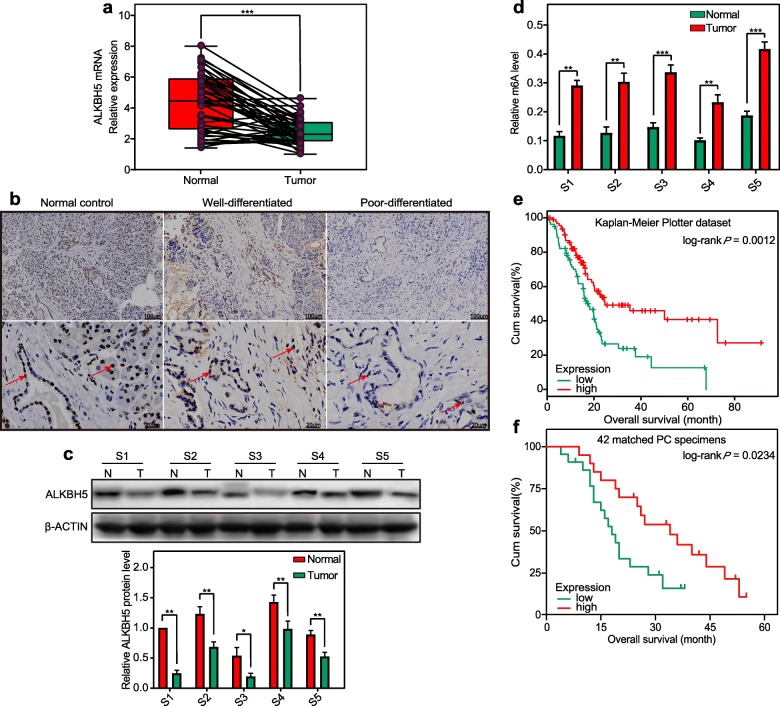
Downregulation of the m6A demethylase ALKBH5 characterizes pancreatic cancer (PC). **a** The ALKBH5 mRNA level demonstrated significantly decreased in PC tissues. **b** Immunohistochemistry staining showed masses of ALKBH5-positively stained cells in normal control tissues and well-differentiated PC tissues, while negative staining in poorly differentiated PC tissues. **c** Western blotting verified the decreased ALKBH5 level in poorly differentiated PC samples (T) when compared to noncancerous controls (N). **d** The elevation of mRNA m6A levels in PC samples depended on the loss of ALKBH5. **e, f** PC patients from the Kaplan-Meier Plotter dataset **e** and 42 matched PC specimens **f** displayed a significant correlation between low ALKBH5 expression (median split) and shorter OS times. A Kaplan-Meier survival curve with log-rank test was applied for prognostic evaluation. Bar graph indicates the means ± SDs of 3 independent experiments. β-actin and GAPDH are used as internal controls in western blotting and qRT-PCR assays, respectively. *P < 0.05, **P < 0.01, and ***P < 0.001

To examine ALKBH5 expression in PC and its conceivable clinical significance, 42 cases of tumour and corresponding noncancerous tissue from PC patients were subjected to investigation. In result, real-time qPCR showed a significant reduction in ALKBH5 mRNA levels in PC tissues compared to noncancerous controls (Fig. 1a). Immunohistochemical staining confirmed the high ALKBH5 level in noncancerous pancreatic tissues, whereas low ALKBH5 levels were observed in most of these PC samples (22/42) (Table 1). Moreover, immunohistochemical staining and western blotting uncovered a significantly decreased expression of ALKBH5 in poorly differentiated PC specimens (11/14), though comparable-high ALKBH5 expression dominated both well- and moderately differentiated PC specimens (Fig. 1b–c). In line with the observation that ALKBH5 loss was associated with increased m6A methylation, we detected significantly enhanced m6A mRNA levels in PC specimens compared to noncancerous controls (Fig. 1d). Survival analysis further identified an association between low ALKBH5 expression and short OS time in 177 PC patients from the Kaplan-Meier Plotter dataset (www.kmplot.com) and 42 matched PC patients in the present study (Fig. 1e, f). Thus, ALKBH5 loss served as a predictive and prognostic indicator of PC. But ALKBH5 level was not filtered to be an independent risk factor of PC as evaluated by the multivariate Cox regression analysis in a cohort of 42 PC patients.

### ALKBH5 exerted transcriptomic impact on PC cells

**Fig. 2:**
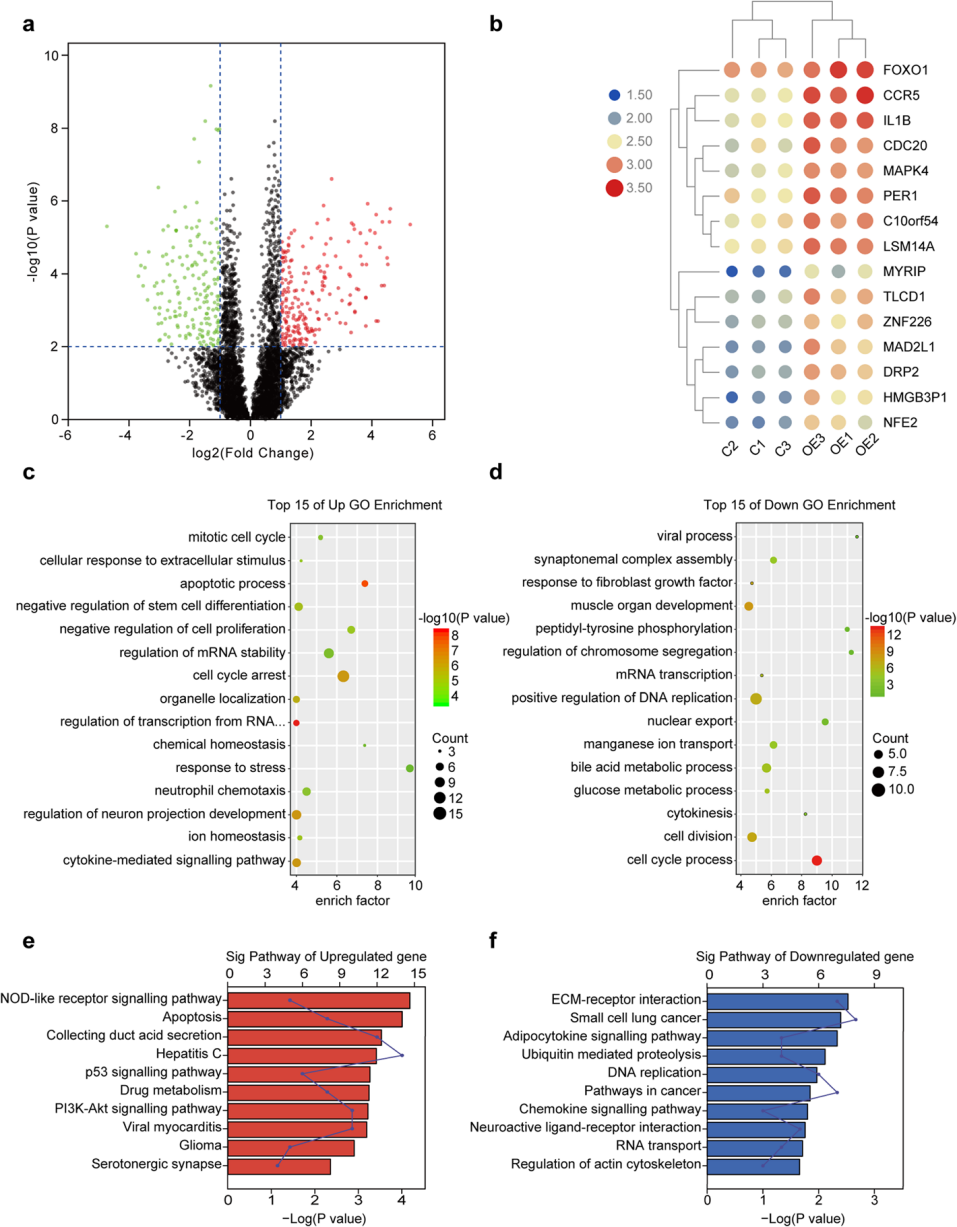
ALKBH5 effects on the transcription profile of PC cells. **a** A volcano plot shows the statistically upregulated (red) and downregulated (green) genes between BxPC-3/vector cells (Control) and BxPC-3/Lv-ALKBH5 cells (ALKBH5 overexpression). **b** Heatmap exhibits the top 15 upregulated genes between BxPC-3/vector **c** and BxPC-3/Lv-ALKBH5 (OE) cells. C: Control group; OE: Overexpression group. **c, d** Gene ontologies (GOs) related to the upregulated **c** and downregulated genes **d** reflected an enrichment of processes concerning cell cycle, apoptosis, and proliferation. **e, f** Signalling pathways related to the upregulated (e) and downregulated genes **f** uncovered a role for differentially expressed genes in signal transduction

When compared to that of BxPC-3/vector cells, RNA-Seq analysis revealed a total of 367 differentially expressed genes (204 upregulated genes, 163 downregulated genes) in ALKBH5-overexpressing PC cells (Fig. 2a,) (supplementary Table 2). Then, unsupervised hierarchical clustering identified the top 15 upregulated genes, such as PER1, FOXO1, CDC20, and CCR5 et al. (Fig. 2b). Furthermore, GO analysis filtered multiple terms associated with cell cycle arrest, anti-proliferation, and apoptosis (e.g., cell cycle arrest, negative regulation of proliferation, apoptotic process) in specific upregulated genes in ALKBH5-overexpressing PC cells (Fig. 2c). In contrast, GO terms involved in cell survival and proliferation demonstrated significant downregulation after ALKBH5 overexpression (Fig. 2d). Total unigenes were then assigned to pathway visualization in an unbiased fashion according to the KEGG database. The PI3K-AKT and P53 signalling pathways, which exhibit close relationships to cell cycle and growth, reflected the high-ranking upregulated pathways. Whereas DNA replication, pathways in cancer and small cell lung cancer were the most downregulated pathways (Fig. 2e, f). Taken together, ALKBH5 displayed PC-suppressive activity on the basis of transcriptome regulation.

### ALKBH5 inhibited proliferation, migration and invasion of PC cells

To determine the role of ALKBH5-dependent m6A demethylation, we inquired into the ALKBH5-regulated PC cells. First, ALKBH5 expression was assessed in normal pancreatic ductal epithelial cells (HPDE6c7) and PC cell lines (SW1990, AsPC-1, CFPAC-1, Panc-1 and BxPC-3). Then, BxPC-3 cells with the lowest level of ALKBH5 expression were transfected with an ALKBH5-encoding lentivirus (BxPC-3/Lv-ALKBH5 group) and empty vectors (BxPC-3/vector group), respectively. SW1990 cells exhibited the highest level of ALKBH5 were treated with the lentivirus encoding a short hairpin RNA specific for ALKBH5 (SW1990/shALKBH5 group), resulting in approximately 90% reduction of its level when compared to that of scrambled (SW1990/scramble group) and untreated controls (SW1990 group) (Supplementary Fig. S1).

**Fig. 3:**
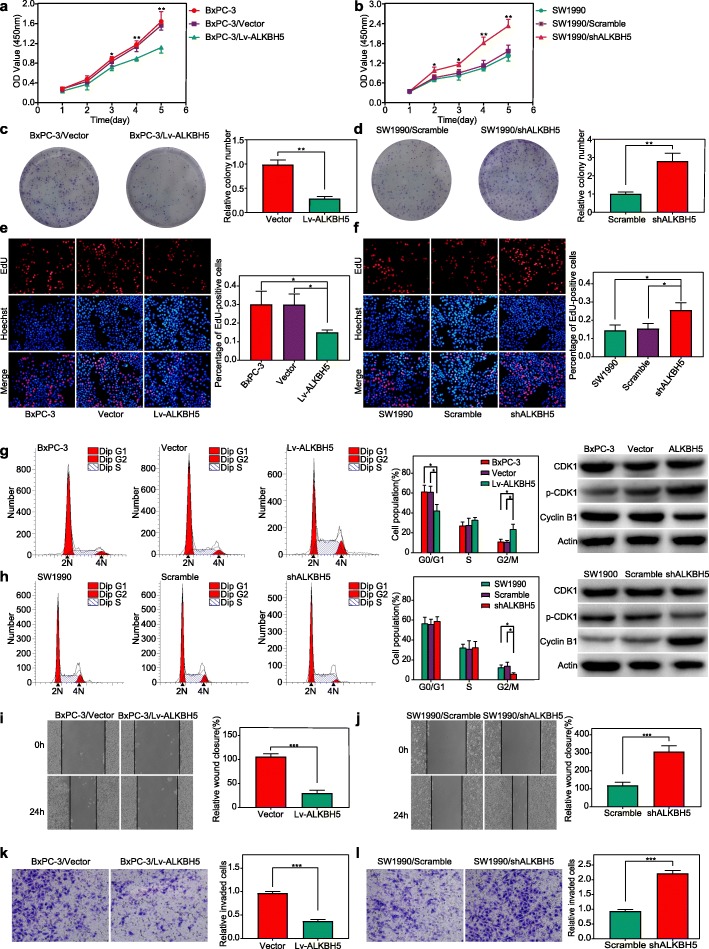
ALKBH5 attenuation of proliferation, cell cycle, migration and invasion of PC cells. ALKBH5 overexpression significantly decreased the proliferation rate **a, e** and weakened the colony-formation ability **c** of BxPC-3 cells, whereas ALKBH5 knockdown promoted SW1990 cell growth, as measured by the CCK-8, EdU **b, f** and colony formation **d** assays. Flow cytometry combined with western blotting revealed that ALKBH5 overexpression (Lv-ALKBH5 group) induced remarkable increase in the percentage of BxPC-3 cells in the G2/M phase, and lead to Cyclin B1 decrease and p-CDK1 increase **g**. Significantly decreased cell number in the G2/M phase with Cyclin B1 increase and p-CDK1 decrease was observed in condition of ALKBH5 knockdown (shALKBH5 group) **h**. Both wound healing and transwell assays showed the suppressed migration **i** and invasion **k** of PC cells, respectively, following ALKBH5 overexpression. In contrast, ALKBH5 knockdown promoted both migration (wound closure) **j** and invasion **l** of PC cells. *P < 0.05, **P < 0.01, ***P < 0.001

Interestingly, ALKBH5 overexpression led to a decreased cell proliferation rate in the BxPC-3/Lv-ALKBH5 group compared to that of the BxPC-3/vector and untreated control (BxPC-3) groups (Fig. 3a, e). In contrast, ALKBH5 knockdown in the SW1990/shALKBH5 group resulted in a marked increase in the cell proliferation rate when compared to that of the SW1990/scramble and untreated control (SW1990) groups (Fig. 3b, f). A colony formation assay was also employed to determine the long-term impact of ALKBH5 on the PC cell proliferation. We observed fewer colonies in the BxPC-3/Lv-ALKBH5 group than in the BxPC-3/vector group after 2 weeks (Fig. 3c), whereas increased colony formation characterized the SW1990/shALKBH5 group (Fig. 3d). A flow cytometry assay further demonstrated G2/M arrest in the BxPC-3/LV-ALKBH5 group (Fig. 3g), and a decreased proportion of cells in the G2/M phase were observed after ALKBH5 knockdown (SW1990/shALKBH5 group) (Fig. 3h). The checkpoint involved in the G2/M phase is controlled by the Cyclin B1/CDK1 complex. We also found a decreased expression of Cyclin B1 and increased expression of p-CDK1 in the BxPC-3/LV-ALKBH5 group (Fig. 3g) (Fig. S3a). However, ALKBH5 knockdown (SW1990/shALKBH5 group) significantly increased the expression of Cyclin B1 and decreased the expression of p-CDK1 (Fig. 3h) (Fig. S3b). Finally, ALKBH5 overexpression in BxPC-3 cells inhibited wound closure and invasive activity (Fig. 3i, k), whereas promotion of wound closure and invasiveness were observed in ALKBH5-silenced SW1990/shALKBH5 cells (Fig. 3j, l).

### ALKBH5 suppressed PC growth and metastasis

**Fig. 4:**
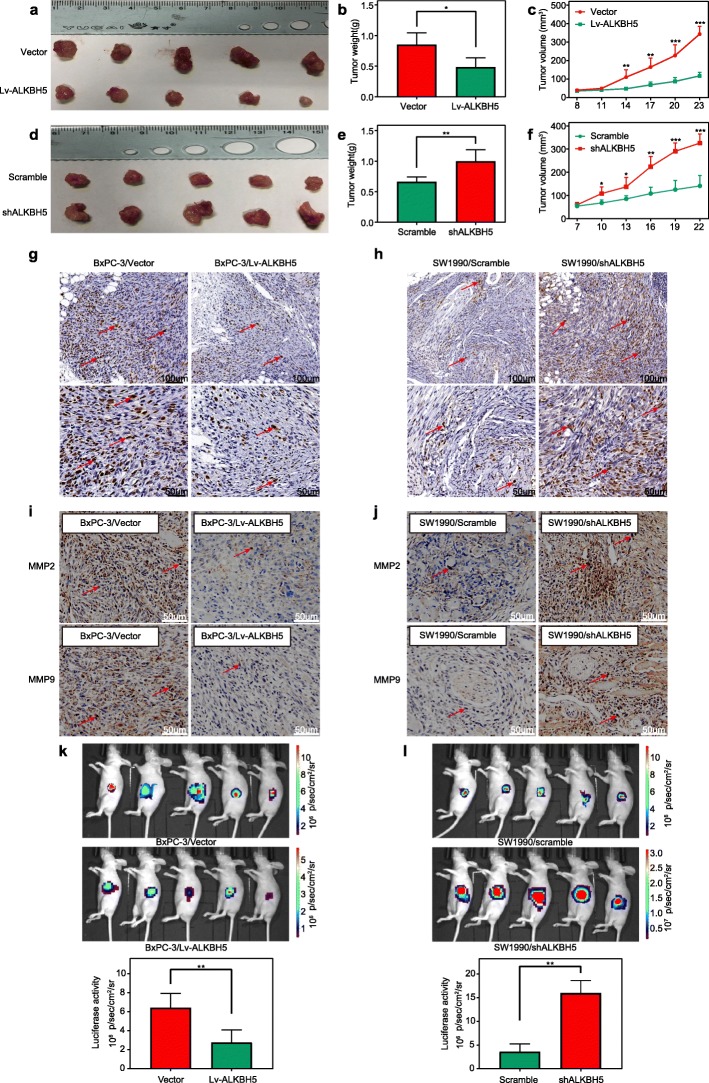
ALKBH5 inhibits tumour growth and invasion potential of PC models. **a-c** Xenografts derived from BxPC-3/Lv-ALKBH5 cells (ALKBH5 overexpression) demonstrated tumour weight **b** and volume **a, c** that were much lower than those of BxPC-3/vector-derived ones (empty). **d**-**f** However, xenografts generated from SW1990/shALKBH5 cells (ALKBH5 knockdown) exhibited a significant increase in tumour weight **e** and volume growth **d, f** when compared to those of SW1990/scramble-generated tumours (nude). **g**-**h** Immunohistochemical labelling showed the downregulated expression of ki-67 after ALKBH5 overexpression **g** and the upregulation of ki-67 after ALKBH5 knockdown **h**. **i-j** Immunohistochemical labelling showed the downregulated expression of MMP-2 and MMP-9 after ALKBH5 overexpression **i** and the upregulation of both MMPs after ALKBH5 knockdown **j**. **k-l** Luciferase activities verified the decreased and increased tumour growth, respectively, in orthotopic PC models established by BxPC-3/Lv-ALKBH5 cells **k** and SW1990/shALKBH5 cells **l**. *P < 0.05, **P < 0.01, and ***P < 0.001

To verify the effect of ALKBH5 on PC, ALKBH5-modified BxPC-3 and SW1990 cells were subcutaneously implanted into the right flank of nude mice, respectively. When compared to PC cells with low-level ALKBH5 expression (BxPC-3/vector group, SW1990/shALKBH5 group), we found a dramatically decreased tumour volume in groups of the BxPC-3/Lv-ALKBH5 and SW1990/scramble (Fig. 4a, c, d, f). In similar, significantly lower tumour weight was detected in the PC xenograft model derived from BxPC-3/Lv-ALKBH5 group when compared to that of the BxPC-3/vector group (Fig. 4b) and in the PC xenograft model derived from SW1990/scramble instead of SW1990/shALKBH5 cells (Fig. 4e). In vivo expression of Ki-67, one of the key indicators of cell growth, was also decreased in the BxPC-3/Lv-ALKBH5 and SW1990/scramble groups in comparison to that of BxPC-3/vector and SW1990/shALKBH5 group with low-level ALKBH5 expression (Fig. 4g, h).

Matrix metalloproteinase 2 (MMP-2) and MMP-9, which indicate the invasive and metastatic capabilities, were simultaneously measured in PC tissues. The limited intensity of MMP-2 and MMP-9 staining in the BxPC-3/Lv-ALKBH5 group revealed a much lower expression of MMPs than what was observed in the BxPC-3/vector group (Fig. 4i). However, ALKBH5 knockdown in the SW1990/shALKBH5 group led to the immunohistochemical signals of MMP-2 and MMP-9 much higher than those in the SW1990/scramble group (Fig. 4j).

Furthermore, we developed an orthotopic PC model to highlight the effect of ALKBH5 using a bioluminescence imaging (BLI) system. Consistent with the findings from the xenograft PC model, the nude mice implanted with BxPC-3/Lv-ALKBH5 cells exhibited significantly lower luciferase activity than those exposed to BxPC-3/vector cells (Fig. 4k). In contrast, the luciferase signal of mice bearing SW1990/shALKBH5 cells was much higher than that of mice with the SW1990/scramble cells (Fig. 4l).

### ALKBH5 targeted PER1 in PC regulation

**Fig. 5:**
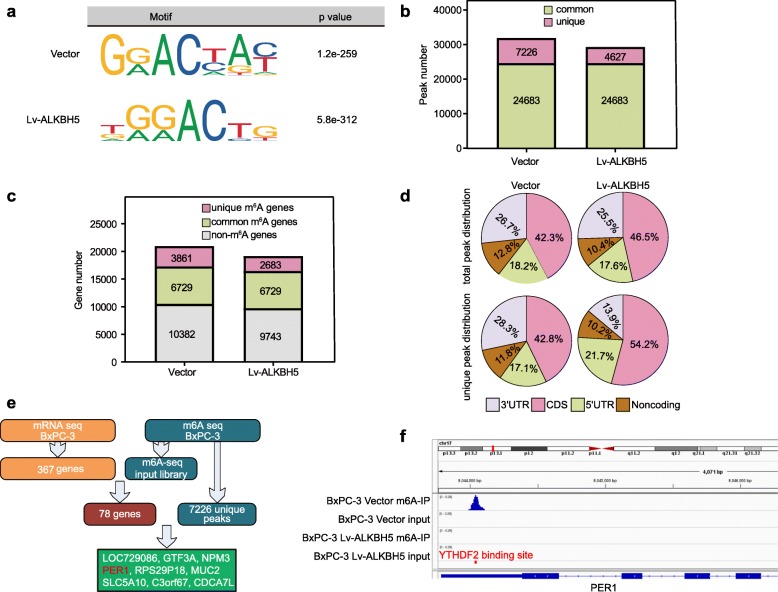
m6A methylation underlies the effects of ALKBH5. **a** Motif analysis using the program HOMER identified “GGACT” as the m6A consensus motif of BxPC-3 cells. **b** m6A-seq determined the number of m6A peaks in PC cells in the vector and Lv-ALKBH5 BxPC-3 groups. **c** m6A-seq determined the number of m6A-modified transcripts in both groups. **d** Distribution of total and unique m6A peaks in both groups is shown. **e** Bioinformatic analysis filtered PER1 as a downstream target of ALKBH5. **f** An m6A modification site in the 3′-UTR of PER1 mRNA was close to the YTHDF2 binding site. ALKBH5 overexpression abolished m6A modification of PER1 mRNA in BxPC-3 cells

After RNA-seq analysis of ALKBH5 overexpression BxPC-3 cells (BxPC-3/Lv-ALKBH5) and controls (BxPC-3/vector), we found a decrease in the global m6A levels of Lv-ALKBH5 cells (Supplementary Fig. S2). Next, we conducted m6A-Seq to map the m6A modification. The m6A consensus motif of GGACT was presented with high enrichment in the BxPC-3 cells (Fig. 5a). In total, m6A-seq identified 31,909 and 29,310 m6A peaks from 10,590 and 9412 m6A-modified transcripts in vector (normal control) and Lv-ALKBH5 cells, respectively (Fig. 5b, c). In contrast to unique m6A peaks in the groups of Lv-ALKBH5 (4,627) and vector (7226), there were 24,683 common peaks of m6A modification in both groups (Fig. 5b). Further investigation of the m6A peak distribution indicated a similar pattern of total m6A distribution in the vector and Lv-ALKBH5 groups. The 7226 unique peaks, which were supposed to contain the target genes of ALKBH5, showed a relatively increased m6A abundance in the 3′UTR of mRNAs in the vector group (Fig. 5d). Intersecting the m6A input RNA-seq dataset and 367 differentially expressed genes obtained by RNA sequencing, a total of 78 genes were defined to be relevant to the ALKBH5 effect. Next, filtering the 78 genes with the 7226 unique m6A peaks resulted in a set of 9 genes: LOC729086, GTF3A, NPM3, PER1, RPS29P18, MUC2, SLC5A10, C3orf67, and CDCA7L (Fig. 5e). In the BxPC-3/vector cells, peak calling analysis distinguished an m6A peak enrichment in the 3′-UTR of PER1 mRNA that was diminished upon ALKBH5 overexpression (Fig. 5f).

### ALKBH5 loss downregulated PER1 mRNA levels in an m6A-YTHDF2-dependent manner

**Fig. 6:**
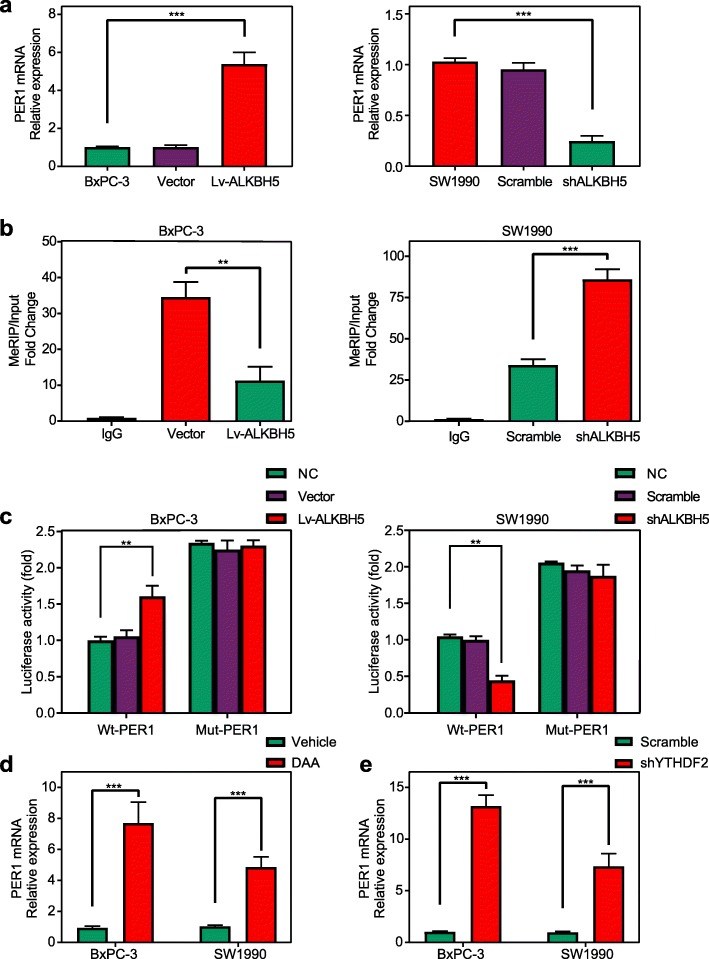
ALKBH5 increases PER1 mRNA levels in an m6A-YTHDF2-dependent manner. **a** ALKBH5 overexpression upregulated the mRNA level of PER1, whereas ALKBH5 knockdown decreased its mRNA level. **b** MeRIP-qPCR analysis confirmed that ALKBH5 overexpression depleted the m6A modification of PER1 mRNA, while the m6A modification of PER1 mRNA was enriched upon ALKBH5 knockdown. **c** Luciferase assays showed that ALKBH5 overexpression or mutation of m6A consensus site in PER1 mRNA relieved the posttranscriptional repression of PER1, but ALKBH5 knockdown promoted PER1 repression. **d** Treatment with a global methylation inhibitor (DAA) led to the upregulation of PER1 mRNA levels in BxPC-3 and SW1990 cells. **e** Knockdown of an m6A-specific RNA binding protein (YTHDF2) increased the PER1 mRNA levels in both cells. **P < 0.01, ***P < 0.001

BxPC-3 cells overexpressing ALKBH5 (BxPC-3/Lv-ALKBH5) demonstrated significant upregulation of PER1 transcripts, whereas ALKBH5 knockdown in SW1990 cells (SW1990/shALKBH5) resulted in a reduction of PER1 mRNA levels (Fig. 6a).

MeRIP-qPCR was then applied to confirm the ALKBH5-mediated m6A demethylation of PER1 mRNA. When compared to the IgG group (pulldown control), an enrichment of PER1 mRNA was obtained by the reaction to m6A-specific antibody. As expected, ALKBH5 overexpression markedly reduced the m6A level of PER1 mRNA. However, the m6A level of PER1 mRNA experienced a significant increase after ALKBH5 knockdown (Fig. 6b).

Thereafter, we replaced N6-methylated adenosine (A) with C (cytosine) in the m6A consensus sequence of PER1 mRNA to establish a mutant PER1 that resists to m6A modification. In the luciferase assay, both BxPC-3 and SW1990 cells transfected with the mutant PER1-fused reporter exhibited PER1 mRNA levels that were increased over the levels observed in the cells treated with the wild-type PER1 (Wt-PER1 group). On the other hand, ALKBH5 overexpression and knockdown resulted in the increase and decrease of luciferase activity, respectively, with the existence of wild-type PER1-fused reporter (Fig. 6c). Thus, ALKBH5 was found to regulate the mRNA level of PER1.

Administration of a global methylation inhibitor, 3-deazaadenosine (DAA), substantially increased the PER1 mRNA level in PC cells (Fig. 6d). In addition, the m6A modification site in the PER1 mRNA located in close to the YTHDF2 binding site (Fig. 5f). Our experiments also showed the ascent PER1 mRNA levels in BxPC-3 and SW1990 cells upon YTHDF2 knockdown (shTYHDF2 group) (Fig. 6e). Thus, ALKBH5 is thought to increase PER1 mRNA levels by abolishing m6A-YTHDF2-dependent mRNA degradation.

### PER1 served as a suppressor of PC

**Fig. 7:**
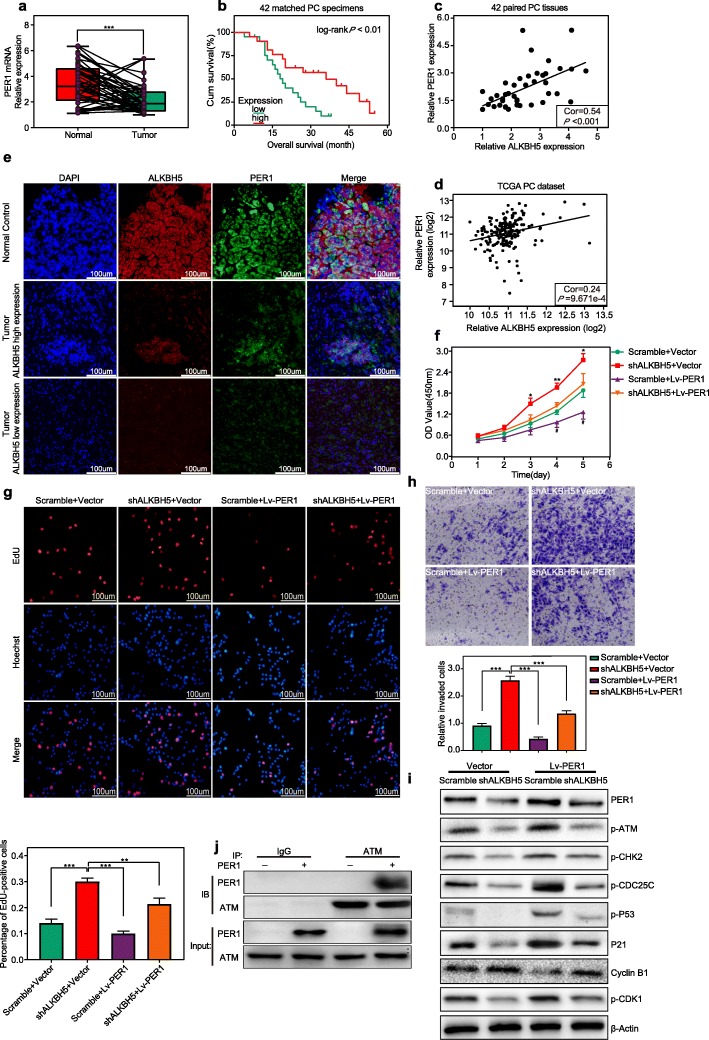
ALKBH5 is correlated with PER1 expression in PC tissue with a tumour suppressive effect. **a** PER1 was significantly downregulated in PC samples. **b** Kaplan-Meier survival curve with log-rank test was applied to 42 matched PC patients. The prognostic evaluation displayed a significant correlation between low PER1 expression (median split) and shorter OS times. **c-d** ALKBH5 correlated with PER1 expression in both our PC cohort **c** and TCGA PC dataset **d**. **e** Immunofluorescent staining demonstrated the expression correlation of ALKBH5 and PER1 and their colocalization in PC samples. **f-h** PER1 overexpression inhibited PC progression and reduced the proliferative and invasive activity of PC cells upon ALKBH5 knockdown. **i** ALKBH5 knockdown resulted in the downregulation of genes promoting G2/M phase cell cycle arrest and upregulation of CYCLIN B1 on a basis of PER1-loss, whereas PER1 overexpression partially reversed these abnormalities. **j** A PER1 overexpression plasmid or an empty plasmid was transfected into 293 T cells. CoIP demonstrated that ATM and PER1 could be coprecipitated. *: shALKBH5 + vector VS shALKBH5 + Lv-PER1, ^#^: scramble+vector VS scramble+Lv-PER1, *P < 0.05, **P < 0.01, ***P < 0.001, and ^#^*P* < 0.05

In the present study, we validated PER1 downregulation in 42 paired PC tissues (Fig. 7a). Survival analysis further identified a prominent association between low PER1 expression and short OS time in 42 matched PC patients (Fig. 7b). Notably, PER1 expression was positively correlated with the ALKBH5 level in the PC cohorts of the present study and the TCGA PC dataset (Fig. 7c, d). Their coincident high-level, and low-level expression was immunofluorescently observed no matter in the normal and PC tissues (Fig. 7e). Furthermore, CCK-8, EdU and transwell assays validated that ectopic expression of PER1 suppressed PC progression and partially rescued the abnormalities observed in SW1990 cell proliferation and invasion following ALKBH5 knockdown (Fig. 7f, g, h). Mechanistically, we found that normalizing PER1 levels led to the reactivation of ATM-dependent signalling with upregulated levels of p-ATM, p-CHK2, p-CDC25C, p-P53, P21, and p-CDK1 and reduced levels of CYCLIN B1 in SW1990/shALKBH5 cells (Fig. 7i) (Fig. S4). The coprecipitation of ATM and PER1 in CoIP experiments substantially substantiated their interaction (Fig. 7j).

### PER1-induced restoration of P53 upregulated ALKBH5 transcription

**Fig. 8:**
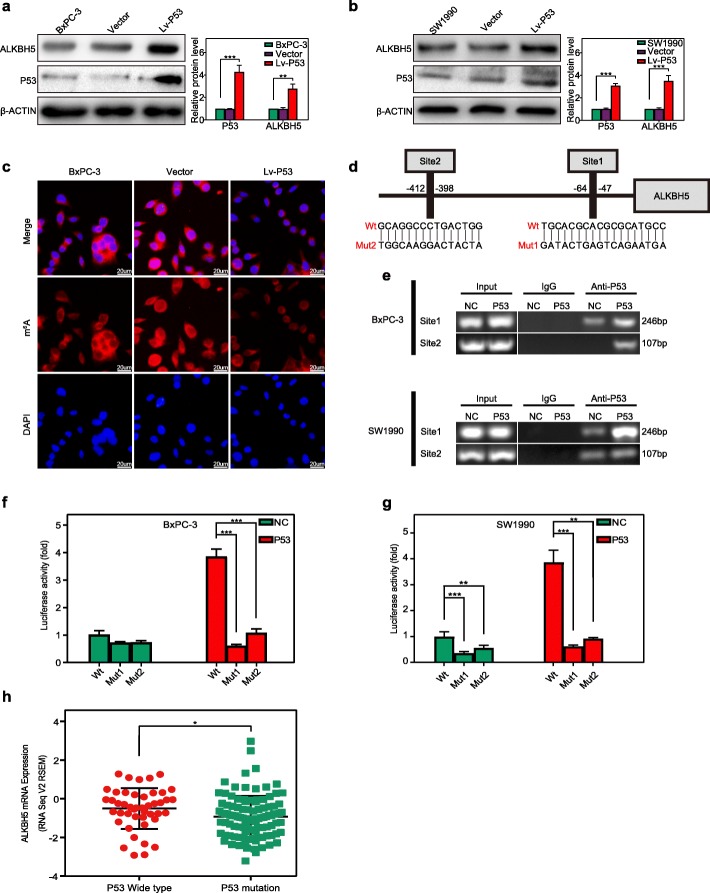
PER1-induced P53 activates ALKBH5 transcription. **a, b** P53 overexpression induced the upregulation of ALKBH5 in both BxPC-3 **a** and SW1990 cells **b**. **c** P53 overexpression reduced the m6A level of total RNA in BxPC-3 cells. **d** A schematic diagram illustrates two potential P53-binding sites and corresponding mutants in the ALKBH5 promoter. **e** ChIP and PCR analysis of BxPC-3 and SW1990 cells identified the binding of P53 and 2 sites in the ALKBH5 promoter. DNAs were pull-downed by a P53 or IgG antibody. The input was used as an internal positive control. **f, g** Luciferase assay for the wild-type or mutant sequence of ALKBH5 binding sites verified the activity of sites 1 and 2 in BxPC-3 and SW1990 cells with (red) or without (green) ectopic P53 expression. **h** PC samples from the P53 wild-type group exhibited ALKBH5 expression levels that were much higher than those of the P53 mutation group. *P < 0.05, **P < 0.01, and ***P < 0.001

As a result of ectopic P53 expression, a significant increase in ALKBH5 levels was documented in both BxPC-3 and SW1990 cells (Fig. 8a, b). Immunofluorescent staining revealed that P53 expression reduced the m6A levels in the total RNA of BxPC-3 cells (Fig. 8c). Being identified by the genomic analysis, two P53-binding domains in the ALKBH5 promoter shed light on the mechanisms underlying P53’s effect (Fig. 8d). Then, we designed two primer sets covering P53-binding sites and performed the Chip assay in PC cells transfected with a P53 expression vector or an empty control. P53 was resultantly proved to bind to both sites, with a majority bound at site 1 (Fig. 8e). A luciferase assay further confirmed that P53 stimulated the expression of ALKBH5 with a wild-type promoter, while this effect was abrogated by a mutation at site 1 or site 2 (Fig. 8f, g). Analysis of TCGA datasets also revealed high ALKBH5 levels in the P53 wide-type group and low ALKBH5 levels in the P53 mutation group (Fig. 8h). Therefore, P53 is shown to activate the ALKBH5 transcription by binding to its promoter.

## Discussion

M6A is well described as the most abundant internal chemical modification of human mRNA. Owing to recently developed high-throughput sequencing technology and m6A-specific antibodies, researchers can precisely determine exact m6A sites and further unravel its functions in both biological and pathological processes [23]. Accumulating evidence indicates that m6A modifications are implicated in various solid tumours [31–34]; however, thus far, there are few studies on m6A distribution and its specific underlying role and mechanisms in PC. In the present study, we revealed that ALKBH5 mRNA and protein levels were significantly downregulated in PC tissues compared to matched noncancerous tissues. This finding is consistent with a previous cytological study in PC cells [35], but it is opposite to several reports about the increased ALKBH5 expression in some other tumours [20, 36]. A plausible explanation for this paradox might be the tissue-specific expression of ALKBH5, and we suppose that ALKBH5 holds discriminant function in different tumour types by intricate mechanisms with respect to tumourigenesis. Furthermore, by referring to the information collected on patients’ clinicopathological characteristics, our immunohistochemistry stanning results proved that low expression of ALKBH5 significantly affected TNM stage, tumour differentiation, and vascular invasion of PC. Our further Kaplan-Meier survival analysis demonstrated that low ALKBH5 expression leads to worse OS in PC patients, which is consistent with a previous retrospective multicohort study based on the analysis of deposited PC data from International Cancer Genome Consortium (ICGC) and The Cancer Genome Atlas (TCGA) databases [37]. These aforementioned findings pave new ways for the clinical use of the m6A demethylase gene (ALKBH5) as a biomarker for the prediction of PC initiation and progression, as a favourable survival outcome prognostic factor in patients, and as a sensitive therapeutic target for future anti-tumour drug development.

To further illuminate the influence of ALKBH5 on the proliferative responses and invasive activity of PC cells, we performed an RNA-seq assay and found significant transcriptome alterations in which the differentially upregulated expressed genes involved in cell cycle arrest, apoptotic process and negative regulation of cell proliferation after ALKBH5 overexpression in BxPC-3 cells. Hence, the profiling prompted a tumour suppressor image of ALKBH5. Furthermore, in vitro and in vivo studies confirmed the inhibitory effect of ALKBH5 on PC cell proliferation and invasion. Next, by RNA-seq and m6A-seq analysis, we identified PER1 as a downstream target of ALKBH5-mediated m6A modification, which was further convinced by the MeRIP-qPCR analysis, luciferase and qRT-PCR assays in control and ALKBH5-overexpression/knockdown PC cells. Circadian misalignment originating from changes in genetic background and behaviour (e.g., shift work and late evening activities) or metabolic modifications (e.g., obesity and diabetes) has been strongly linked to tumour pathologies [38–40]. PER1, one of the core circadian genes, plays a critical role in the control of mammalian circadian rhythms, cell cycle, and DNA damage response [40–43]. Previous studies have also reported that aberrant expression and dampened rhythm of PER1 intimately linked to the inception and progression of malignant tumours, such as colon cancer, prostate cancer, breast cancer and non-small cell lung cancer (NSCLC) [42, 44–46]. Furthermore, mechanistic exploration indicates an interaction between PER1 and ataxia-telangiectasia-mutated (ATM) [41]. ATM, acting as a sensor of DNA damage, regulates the CYCLIN B1/CDK1 complex by targeting ATM-CHK2-P53/CDC25C signalling, and then performs an essential role in DNA repair and cell cycle checkpoint arrest [47–49]. Consistently, PER1 overexpression and subsequent activation of downstream ATM-Chek2 signalling has been proved to suppress the growth of multiple human cancer cell lines by inducing G2/M cell cycle arrest [40]. In this study, we demonstrated the downregulation of PER1 in PC, which is in accord with the findings of previous reports [50, 51]. However, the pathogenic mechanism underlying PER1 downregulation in PC tumourigenesis remains yet unknown.

The direct binding between PER1 mRNA and YTHDF2 has previously been revealed by YTHDF2 PAR-CLIP-Seq analysis [15]. According to the results of the present study, the defined YTHDF2 binding site is located around the m6A enrichment regions in the 3′ UTR. We also demonstrated a significantly augmented PER1 mRNA level in both BxPC-3 and SW1990 cells upon the knockdown of YTHDF2. Thus, multiple lines of evidence sustained our notion that the PER1 transcript is directly targeted by YTHDF2, and decreased ALKBH5 expression leads to the PER1 silence through an m6A-YTHDF2-dependent manner.

To gain deeper insight into the influence of dysregulated ALKBH5-m6A-YTHDF2-PER1 axis in PC, we performed PER1 rescue experiments in SW1990 cells with ALKBH5 knocked down. The suppression of the rates of cell proliferation and invasion indicated a PER1-dependent anti-tumour function of ALKBH5 in PC progression. Meanwhile, western blotting showed that molecules involved in the ATM-dependent pathway (p-ATM (Ser-1981), p-CHK2 (Thr-68), p-CDC25C (Ser-216), p-P53 (Ser-15), P21 and p-CDK1 (Tyr-15)) were decreased in shALKBH5 PC cells and could be restored by the overexpression of PER1. Cyclin B1 simultaneously showed the opposite expression pattern. To further explain the potential mechanisms by which PER1 regulates the ATM-dependent signalling pathway, we performed CoIP experiments and demonstrated a direct interaction between PER1 and ATM. Thus, we suggest that PER1 is necessary for the activation of ATM or may serve as an adaptor protein recruiting other substrates to ATM. Collectively, we indicated that ALKBH5 loss induced PER1 inhibition in PC cells with a result of cell cycle progression from G2 to M phase on the control of the CYCLIN B1/CDK1 complex, and PER1 restoration suppressed proliferation of PC cells by activating ATM-dependent signalling.

Dysregulation of ALKBH5 featured various kinds of tumours [20, 36]. In the present study, we also demonstrated decreased ALKBH5 expression in PC. Referring to bioinformatic predication by genomic analysis and validation by Chip and luciferase assays, we confirmed the binding of P53 to the ALKBH5 promoter, which indicates the transcriptional activation of P53 on ALKBH5. Moreover, ectopic P53 expression indeed increased ALKBH5 expression in PC cells. As a well-known transcription activator, P53 mutations/deletion occur in approximately half of malignant diseases. Consulting the PC data in TCGA datasets, we found that the expression of ALKBH5 in the P53 wide-type group was significantly upregulated, while the P53 mutation cohort exhibited downregulated ALKBH5 levels. Therefore, we propose that low/deficient P53 levels account for downregulated ALKBH5 expression and the corresponding m6A-dependent oncogenic potential in PC.

For the sake of limited population, the risk prognostic role of ALKBH5 in PC progress may not be fully unveiled in present study. PC is well described to be a male-predominated disease worldwide [52]. Rodent models of PC are then established in male nude mice to reflect the gender-related characteristics. However, the lacking of female PC model remains a limitation that deserves future investigation to obtain comprehensive view of PC. In the other aspect of in vivo experiments, combinatorial investigation of xenografic and orthotopic PC models has been applied with the purpose of integrating our findings in situ and ex situ. Additionally, special mediums for PC cell lines could be an approach to optimize the cytological experiments [53–55].

**Fig. 9:**
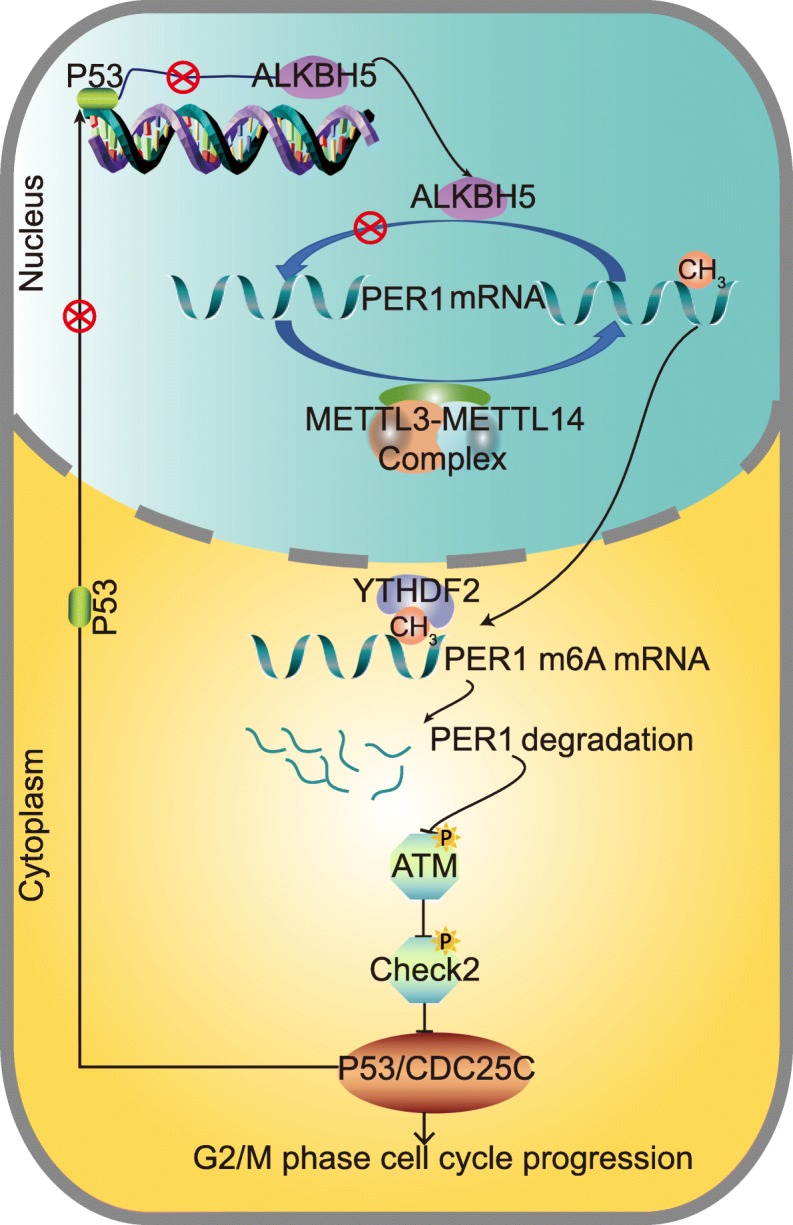
ALKBH5 induces posttranscriptional activation of PER1 to inhibit PC progression in an m6A-YTHDF2-dependent manner. ALKBH5 loss that abrogates mRNA demethylation leads to an upregulation of PER1 m6A levels in PC cells. Then, degradation of PER1 mRNA occurs on the basis of the m6A-YTHDF2 interaction. PER1 downregulation results in the inhibition of ATM phosphorylation and inactivation of the ATM-CHK2-P53/CDC25C pathways. G2/M arrest in PC cells was suppressed as a result. The PER1-related P53 downregulation and P53-dependent transcriptional inactivation of ALKBH5 indicates a feedback loop underlying the progression of PC

In conclusion, ALKBH5 loss is associated with poor clinicopathological characteristics and prognosis of PC. Overexpression of ALKBH5 reduces the cell proliferation, migration, invasion and tumour growth of PC, whereas ALKBH5 knockdown facilitates PC progression. Demethylation of PER1 mRNA and increase in its level underlie the effect of ALKBH5 in an m6A-YTHDF2-dependent manner. PER1-induced P53 upregulation and P53-activated ALKBH5 transcription may reflect a feedback regulation of m6A modifications of PC (Fig. 9).

## Supplementary information

**Additional file 1: Figure S1.** Construction of the ALKBH5-manipulated pancreatic cancer (PC) cell lines. (a) The mRNA expression of ALKBH5 in HPDE6c7 and five PC cell lines. (b) Protein level of ALKBH5 in HPDE6c7 and five PC cell lines. (c) Examination of a stable overexpression of ALKBH5 in BxPC-3 cells (left panel) and stable knockdown of ALKBH5 in SW1990 cells (right panel). **P < 0.01, ***P < 0.001 (t test compared with HPDE6c7 group). **Figure S2.** m6A level in BxPC-3/Vector and BxPC-3/Lv-ALKBH5 cells. Overexpression of ALKBH5 significantly reduced m6A level in BxPC-3 cells.*P < 0.05. **Figure S3.** The semi-quantitative analysis of western blotting results in Fig. 3. (a) ALKBH5 overexpression leads to the upregulated protein level of p-CDK1 and downregulated protein level of Cyclin B1 in BxPC-3cells. (b) The protein level of p-CDK1 and Cyclin B1 was significantly downregulated and upregulated according to the knocking down of ALKBH5 in BxPC-3cells. ***P < 0.001. **Figure S4.** The semi-quantitative analysis of western blotting results in Fig. 7i. ALKBH5 knockdown resulted in the downregulation of genes promoting G2/M phase cell cycle arrest and up-regulation of CYCLIN B1 on a basis of PER1–loss, whereas PER1 overexpression partially reversed these abnormalities. *P < 0.05, **P < 0.01, ***P < 0.001. **Table S1.** Primers used in this study. **Table S2.** 367 differentially expressed genes.

## Data Availability

Please contact the corresponding author for all data requests.

## Abbreviations

CCK-8: Cell Counting Kit-8
chip: Chromatin immunoprecipitation
coip: Co-Immunoprecipitation
EdU: 5-Ethynyl-20-deoxyuridine
EFS: event-free survival
m6A: N6-methyladenosine
OS: Overall survival
PC: Pancreatic cancer
qRT-PCR: Quantitative reverse transcription polymerase chain reaction
