# Supplementary material for: RNA demethylase ALKBH5 prevents pancreatic cancer progression by posttranscriptional activation of PER1 in an m6A-YTHDF2-dependent manner
Source: Mol Cancer. 2020 May 19;19:91. doi: 10.1186/s12943-020-01158-w (PMC7236181; doi:10.1186/s12943-020-01158-w)
Supplement: Supplementary file 1 — Additional file 1: Figure S1. Construction of the ALKBH5-manipulated pancreatic cancer (PC) cell lines. (a) The mRNA expression of ALKBH5 in HPDE6c7 and five PC cell lines. (b) Protein level of ALKBH5 in HPDE6c7 and five PC cell lines. (c) Examination of a stable overexpression of ALKBH5 in BxPC-3 cells (left panel) and stable knockdown of ALKBH5 in SW1990 cells (right panel). **P < 0.01, ***P < 0.001 (t test compared with HPDE6c7 group). Figure S2. m6A level in BxPC-3/Vector and BxPC-3/Lv-ALKBH5 cells. Overexpression of ALKBH5 significantly reduced m6A level in BxPC-3 cells.*P < 0.05. Figure S3. The semi-quantitative analysis of western blotting results in Fig. 3. (a) ALKBH5 overexpression leads to the upregulated protein level of p-CDK1 and downregulated protein level of Cyclin B1 in BxPC-3cells. (b) The protein level of p-CDK1 and Cyclin B1 was significantly downregulated and upregulated according to the knocking down of ALKBH5 in BxPC-3cells. ***P < 0.001. Figure S4. The semi-quantitative analysis of western blotting results in Fig. 7i. ALKBH5 knockdown resulted in the downregulation of genes promoting G2/M phase cell cycle arrest and up-regulation of CYCLIN B1 on a basis of PER1–loss, whereas PER1 overexpression partially reversed these abnormalities. *P < 0.05, **P < 0.01, ***P < 0.001. Table S1. Primers used in this study. Table S2. 367 differentially expressed genes. [file 12943_2020_1158_MOESM1_ESM.pdf]

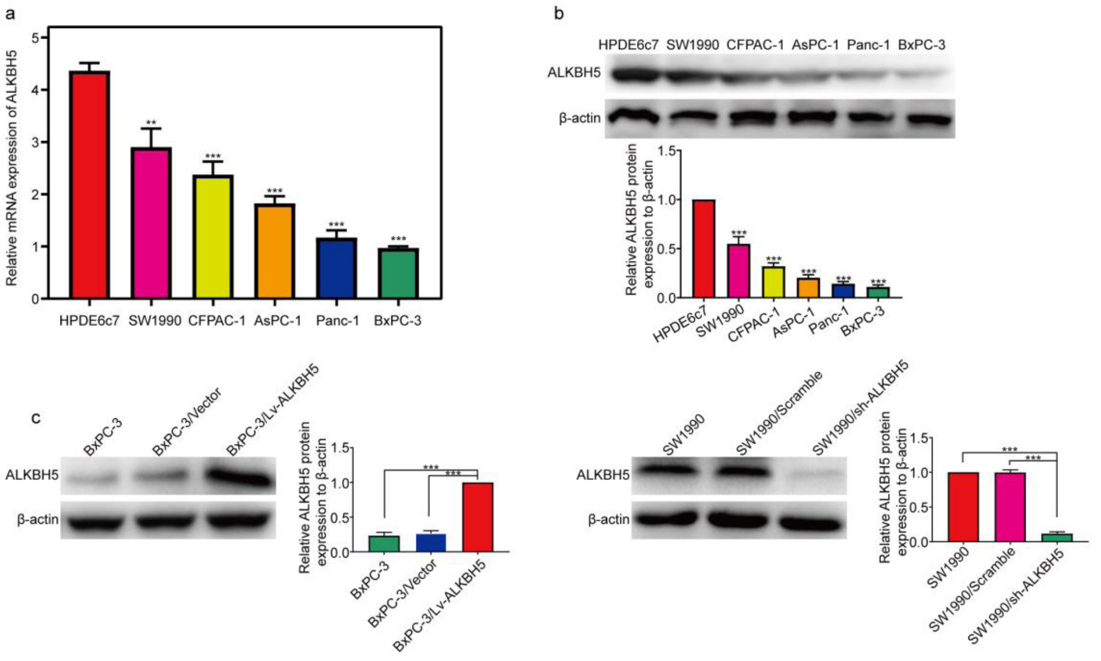

Supplementary Figure S1. Construction of the ALKBH5-manipulated pancreatic cancer (PC) cell lines. (a) The mRNA expression of ALKBH5 in HPDE6c7 and five PC cell lines. (b) Protein level of ALKBH5 in HPDE6c7 and five PC cell lines. (c) Examination of a stable overexpression of LAKBH5 in BxPC-3 cells (left panel) and stable knockdown of ALKBH5 in SW1990 cells (right panel). *P < 0.05, **P<0.01, ***P < 0.001 (t test compared with HPDE6c7 group).

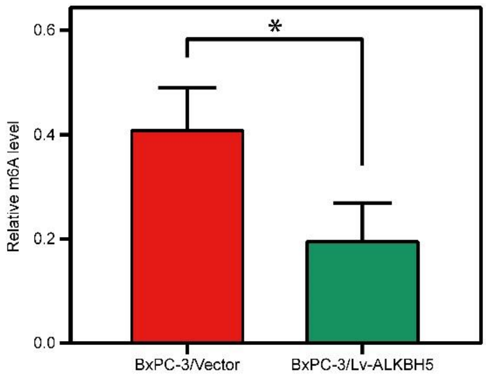

Supplementary Figure S2. m6A level in BxPC-3/Vector and BxPC-3/Lv-ALKBH5 cells. Overexpression of ALKBH5 significantly reduced m6A level in BxPC-3 cells. *P<0.05.

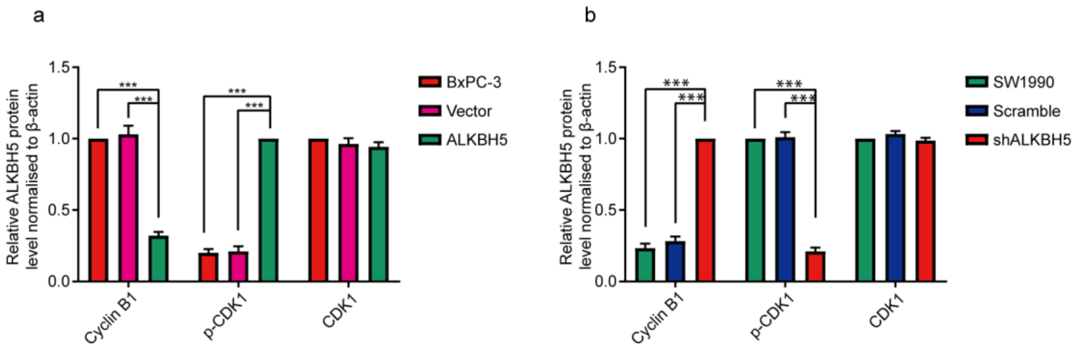

Supplementary Figure S3. The semi-quantitative analysis of western blotting results in figure 3. (a) ALKBH5 overexpression leads to the upregulated protein level of ALKBH5 in BxPC-3 cells. (b) The protein level of ALKBH5 was significantly downregulated according to the knocking down of ALKBH5 in BxPC-3 cells. ***P < 0.001.

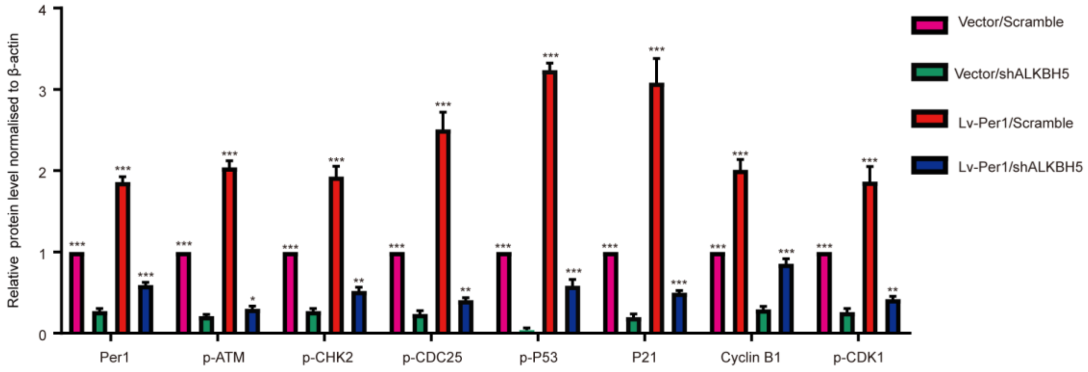

Supplementary Figure S4. The semi-quantitative analysis of western blotting results in figure 7i. ALKBH5 knockdown resulted in the downregulation of genes promoting G2/M phase cell cycle arrest and up-regulation of CYCLIN B1 on a basis of PER1–loss, whereas PER1 overexpression partially reversed these abnormalities. *P < 0.05, **P<0.01, ***P < 0.001.

Supplementary Table S1. Primers used in this study

Gene	Primer sequence (5'-3')	
**qRT-PCR**		
ALKBH5	F: TGAGCACAGTCACGCTTCCC	R: TCCGTGTCCTTCTTTAGCGACTC
PER1	F: AGCAACAGCCACGGTTCTC	R: CAGTCCACACAAGCCATCAC
GAPDH	F: AGTGCCAGCCTCGTCTCATA	R: GGTAACCAGGCGTCCGATA
**MeRIP-qPCR**		
PER1	F: GAGGAATCCTTCCCTCCCCT	R: CTCCATGGTCCACCAACCTC
**Chip**		
Site 1	F: TGAGAGCGTTCCCTTGAAGC	R: GCCCGCACTTTAAAGCTCTC
Site 2	F: ACTGCCTGATTGACACGCAT	R: CCTTTGGCGCTTCCACTTCT

Supplementary Table S2. 367 differentially expressed genes

GeneSymbol	Vector	Lv-ALKBH5	Log2FC	P Value	Style
RPL26L1	726.373	292.941	-1.31	6.72822E-10	down
CSK	342.363	122.122	-1.49	6.42392E-09	down
PRIM1	2683.978	1317.381	-1.03	1.06758E-08	down
WNT6	1472.889	670.704	-1.13	1.07845E-08	down
FAM65B	683.873	327.44	-1.06	1.17193E-08	down
CDCA7	199.261	55.151	-1.85	1.97879E-08	down
CXCL10	667.892	206.84	-1.69	8.60201E-08	down
TOP1MT	136.515	885.848	2.7	2.34423E-07	up
BDKRB2	460.368	56.291	-3.03	4.25305E-07	down
FOS	268.365	78.862	-1.77	1.09977E-06	down
C10orf54	102.511	1505.091	3.88	1.1749E-06	up
FGFR3	237.047	53.647	-2.14	1.45579E-06	down
SREBF2	52.816	280.315	2.41	1.47911E-06	up
MAPK4	9.219	225.129	4.61	1.69824E-06	up
MALT1	2753.733	494.927	-2.48	1.97424E-06	down
MAD2L1	170.554	3100.069	4.18	2.29087E-06	up
ID3	2877.991	1317.737	-1.13	3.08177E-06	down
HSDL2	162.806	988.44	2.6	3.0903E-06	up
ENO3	826.283	256.531	-1.69	3.4578E-06	down
FOXO1	13.829	328.927	4.57	3.80189E-06	up
TLCD1	756.945	15436.357	4.35	3.89045E-06	up
AURKB	637.356	262.024	-1.28	3.95094E-06	down
BBC3	228.635	2294.402	3.33	4.14E-06	up
NR1D2	163.179	754.989	2.21	4.16869E-06	up
HIF1A	4002.376	605.944	-2.72	4.81061E-06	down
NFE2	224.025	8764.864	5.29	5.01187E-06	up
WNT5A	1775.268	67.777	-4.71	5.03037E-06	down
COL3A1	333.461	97.692	-1.77	5.08042E-06	down
FGR	3672.381	852.585	-2.11	5.23118E-06	down
NCF2	159.491	1668.494	3.39	5.24807E-06	up
ZP3	1826.266	480.663	-1.93	5.58342E-06	down
ARID3B	181.617	1922.483	3.4	6.30957E-06	up
THOC7	327.363	44.959	-2.86	6.40324E-06	down
ZW10	255.372	35.072	-2.86	6.40324E-06	down
AREGB	2066.835	377.834	-2.45	6.45952E-06	down
POLR2D	786.399	143.611	-2.45	6.46845E-06	down
FOXO4	35.033	96.747	1.47	6.60693E-06	up
ZNF862	2008.956	5248.648	1.39	7.07946E-06	up
CDC20	129.068	1953.692	3.92	7.07946E-06	up
PER1	165.945	2849.635	4.1	7.58578E-06	up
THBS1	186.276	85.408	-1.13	7.88678E-06	down
ARHGEF9	83.894	1064.157	3.67	7.94328E-06	up

RPS28	2075.026	641.237	-1.69	8.01493E-06	down
PAK6	4537.112	1355.828	-1.74	9.26616E-06	down
NFIX	35.955	148.171	2.04	9.54993E-06	up
S1PR3	83.894	204.505	1.29	9.54993E-06	up
CEBPB	72.831	467.389	2.68	1.12202E-05	up
CCR10	2679.387	207.964	-3.69	1.15452E-05	down
BIRC5	426.511	107.847	-1.98	1.24537E-05	down
CDKN1A	2736.363	1303.111	-1.07	1.34524E-05	down
AKR1C1	1762.893	615.634	-1.52	1.37373E-05	down
TNXB	937.478	371.12	-1.34	1.422E-05	down
CLDN1	32.267	68.807	1.09	1.47911E-05	up
PARP14	59.003	545.985	3.21	1.51356E-05	up
THOC6	887.289	227.995	-1.96	1.65501E-05	down
PRKAA1	33.189	80.624	1.28	1.7378E-05	up
CHD7	44.252	243.989	2.46	1.7378E-05	up
DGKQ	14.751	36.334	1.3	1.86209E-05	up
PCK2	638.982	109.473	-2.55	1.86724E-05	down
TRPC5	25.814	364.578	3.82	1.90546E-05	up
SP6	19.36	64.334	1.73	1.99526E-05	up
HMGB3P1	37.798	754.95	4.32	2.13796E-05	up
IMPDH2	19.36	40.434	1.06	2.29087E-05	up
NEDD9	1552.182	152.058	-3.35	2.43669E-05	down
SYCE2	264.363	108.781	-1.28	2.50265E-05	down
JUNB	43.33	104.532	1.27	2.51189E-05	up
EREG	289.579	140.567	-1.04	2.70583E-05	down
IL1R1	868.386	1968.243	1.18	2.81838E-05	up
PDGFA	2526.889	832.869	-1.6	2.83661E-05	down
FZD4	972.344	142.076	-2.77	2.84708E-05	down
SNORD3B-2	5320.118	389.22	-3.77	2.86946E-05	down
C3orf67	47.94	104.232	1.12	3.0903E-05	up
ZNF296	28.579	152.206	2.41	3.16228E-05	up
STAT5A	3688.351	868.487	-2.09	3.17541E-05	down
BIRC3	621.263	83.915	-2.89	3.22329E-05	down
SPC24	2190.268	436.274	-2.33	3.53834E-05	down
CDK14	3784.221	1649.692	-1.2	3.87079E-05	down
KDM8	3648.625	1590.58	-1.2	3.87079E-05	down
RHBDF1	12.907	159.243	3.63	3.98107E-05	up
IL1A	41.486	175.771	2.08	3.98107E-05	up
PRKAA2	478.977	66.843	-2.84	4.06631E-05	down
EIF3C	1672.273	229.778	-2.86	4.07193E-05	down
KAT2B	14.751	33.411	1.18	4.0738E-05	up
SOD2	35.955	77.205	1.1	4.14E-05	up
CRTC3	36.877	77.019	1.06	4.16869E-05	up
CCR5	16.594	380.721	4.52	4.16869E-05	up

LAMB3	652.387	232.049	-1.49	4.18408E-05	down
ITGA2	1893.733	673.585	-1.49	4.18408E-05	down
APK2	27.657	72.861	1.4	4.2658E-05	up
USP28	11.063	28.25	1.35	4.36516E-05	up
CYLD	27.657	63.078	1.19	4.36516E-05	up
FBXO2	2773.801	467.249	-2.57	4.39845E-05	down
CAMKK1	2678.902	1106.683	-1.28	4.69029E-05	down
IL8	662.384	273.638	-1.28	4.69029E-05	down
KLHL42	827.349	232.527	-1.83	4.80065E-05	down
GSN	852.346	411.542	-1.05	4.85065E-05	down
EIF3F	827.363	399.479	-1.05	4.85065E-05	down
KMT5A	2033.836	4376.275	1.11	5.01187E-05	up
ATP1A1	346.384	52.122	-2.73	5.29541E-05	down
ZNF226	12.907	306.147	4.57	5.49541E-05	up
MYD88	2883.445	607.095	-2.25	5.53223E-05	down
SSR2	198.234	53.329	-1.89	5.62212E-05	down
IL7R	27.657	355.96	3.69	5.62341E-05	up
TRIM22	11.985	168.098	3.81	5.62341E-05	up
MYRF	165.023	991.537	2.59	5.7544E-05	up
ZBTB38	87.582	427.435	2.29	6.0256E-05	up
ARHGAP19	53.243	130.42	1.29	6.30957E-05	up
PDS5A	541.768	1929.527	1.83	6.30957E-05	up
NONO	1428.267	443.426	-1.69	6.41505E-05	down
SYNCRIP	263.511	2069.002	2.97	6.45654E-05	up
ARHGEF2	56.292	258.65	2.2	6.60693E-05	up
HSD17B12	823.615	74.112	-3.47	6.71274E-05	down
CALCOCO2	872.223	387.285	-1.17	6.95665E-05	down
GTF3A	283.398	1435.846	2.34	7.07946E-05	up
SLC12A2	1552.347	371.633	-2.06	7.19283E-05	down
APC	339.352	730.195	1.11	7.4131E-05	up
CYP1B1	673.22	1418.779	1.08	7.94328E-05	up
TOMM22	441.408	2332.997	2.4	8.31764E-05	up
DSG2	2762.363	1005.284	-1.46	8.54673E-05	down
SYNJ1	897.944	2230.238	1.31	8.91251E-05	up
INPP5A	1219.335	4347.224	1.83	9.33254E-05	up
MYH10	341.353	1176.365	1.79	9.54993E-05	up
KMT2D	1007.641	9586.351	3.25	9.54993E-05	up
ATP5H	772.382	121.91	-2.66	0.00010193	down
COL1A2	348.338	119.242	-1.55	0.000104424	down
CYB5R3	2633.736	713.71	-1.88	0.000104448	down
CCT4	961.273	163.938	-2.55	0.000105148	down
PRMT2	771.308	4104.991	2.41	0.000109648	up
RBFA	1086.712	2823.471	1.38	0.000112202	up
ELFN2	49.213	260.65	2.41	0.00011749	up

PMAIP1	430.216	2331.297	2.44	0.00011749	up
SEC61B	862.336	192.459	-2.16	0.000119179	down
RPS3A	4365.166	2103.565	-1.05	0.000119647	down
TARDBP	272.109	574.65	1.08	0.000125893	up
RPL13	655.263	201.346	-1.7	0.000127116	down
TFRC	1782.631	839.503	-1.09	0.000134555	down
CD24	736.871	344.526	-1.1	0.000136553	down
NR1D1	1140.141	2596.759	1.19	0.000141254	up
PABPC3	288.992	39.566	-2.87	0.000150107	down
RAB2A	4629.23	27680.004	2.58	0.000151356	up
CD81	2172.869	387.724	-2.49	0.00015181	down
WASL	18.627	47.073	1.34	0.000162181	up
RGS19	1116.627	5792.344	2.38	0.000165959	up
LSM6	158.206	559.564	1.82	0.000165959	up
NFKBIZ	56.302	137.436	1.29	0.00017378	up
FAU	2837.762	334.054	-3.09	0.000175388	down
AKR1C3	376.124	168.192	-1.16	0.000177174	down
GANAB	20.03	42.594	1.09	0.000177828	up
RPS9	1826.334	631.717	-1.53	0.000181468	down
RAC1	872.234	75.31	-3.53	0.000209508	down
S100A10	1737.372	669.848	-1.38	0.000213255	down
CST3	1802.378	201.898	-3.16	0.000215179	down
CANX	241.08	1250.568	2.38	0.000218776	up
ATP6V1G1	277.826	52.199	-2.41	0.000220242	down
MYRIP	500.881	9866.51	4.3	0.000223872	up
DRP2	14.493	308.106	4.41	0.000223872	up
DIP2C	3601.159	19339.268	2.43	0.000229087	up
YBX1	689.836	258.391	-1.42	0.000234909	down
MIF	683.273	191.994	-1.83	0.000243332	down
PEG10	360.468	873.841	1.28	0.000251189	up
INPP4B	52.312	394.005	2.91	0.000251189	up
CHD2	2496.153	28240.747	3.5	0.00025704	up
CYP1A1	40.261	388.376	3.27	0.00025704	up
VDAC2	284.748	102.249	-1.48	0.000270271	down
LOC729086	36.701	82.496	1.17	0.000275423	up
MAP9	173.047	2098.322	3.6	0.000275423	up
MAPK	92.046	304.286	1.73	0.000281838	up
CHUK	2105.173	10920.286	2.38	0.000281838	up
RRAGC	372.904	1097.618	1.56	0.000288403	up
ARID5A	3082.016	8582.36	1.48	0.000295121	up
LGALS4	476.381	92.583	-2.36	0.000308177	down
SLC6A12	800.271	1967.087	1.3	0.000316228	up
ALDH1A1	428.283	56.926	-2.91	0.000321514	down
GALNT2	3032.367	6906.45	1.19	0.000323594	up

ACO2	5.814	23.811	2.03	0.000323594	up
CAPNS1	2783.746	808.28	-1.78	0.000325462	down
CHIA	336.983	138.183	-1.29	0.00032802	down
CCNI	1067.373	128.841	-3.05	0.000337987	down
BIRC6	63.522	136.967	1.11	0.000338844	up
IFI27	2637.331	500.097	-2.4	0.000339469	down
PSME1	4001.738	1279.719	-1.64	0.000353509	down
ANKRD28	294.83	1242.25	2.08	0.000354813	up
GCH1	2562.604	5498.776	1.1	0.000371535	up
CUL5	8605.209	53084.186	2.63	0.000380189	up
NPM3	862.026	8373.34	3.28	0.000380189	up
PHB2	972.369	455.738	-1.09	0.00039473	down
COX6C	1274.947	604.929	-1.08	0.000421697	down
SDCBP	1638.792	590.182	-1.47	0.000424522	down
SLC5A10	1496.574	21431.54	3.84	0.000436516	up
EPAS1	26.781	380.866	3.83	0.000446684	up
MUC5AC	827.347	202.285	-2.03	0.000448539	down
AKT3	125.893	355.463	1.5	0.000457088	up
ABCG1	3906.926	8947.806	1.2	0.000467735	up
S100A6	347.182	33.205	-3.39	0.000488203	down
COX7A2	872.364	414.143	-1.07	0.000499229	down
RC3H1	218.418	664.418	1.61	0.000501187	up
ALDH1L2	512.923	2473.946	2.27	0.000524807	up
ALDH2	892.263	140.315	-2.67	0.000531496	down
EFCAB6	56.404	128.732	1.19	0.000562341	up
PDE5A	126.435	1045.699	3.05	0.000562341	up
IGFBP2	2648.374	987.811	-1.42	0.000567283	down
CCDC72	2673.213	1184.415	-1.17	0.000572137	down
RPL35	782.4374	271.335	-1.53	0.000586543	down
ALDOA	1738.893	346.126	-2.33	0.000605899	down
RPS29P18	627.959	1344.661	1.1	0.000630957	up
UQCRQ	187.162	88.019	-1.09	0.00065887	down
SIK1	431.114	4246.109	3.3	0.000660693	up
ECM1	482.721	1779.857	1.88	0.000691831	up
LMNB1	93.284	887.472	3.25	0.000691831	up
MAF	1892.326	5089.982	1.43	0.000707946	up
ZNF579	528.323	1710.588	1.7	0.00074131	up
HLA-DPB1	218.637	69.32	-1.66	0.000806121	down
EML4	94.369	196.004	1.05	0.000812831	up
IL26	2075.384	262.589	-2.98	0.000826799	down
RPS27A	689.167	77.979	-3.14	0.000835795	down
LYZ	518.238	237.663	-1.12	0.000852119	down
UQCR10	2783.862	1061.267	-1.39	0.00085487	down
KRT18	652.389	185.733	-1.81	0.00086856	down

CDKN1B	492.454	1124.716	1.19	0.000870964	up
GPNMB	339.511	1032.778	1.61	0.000870964	up
HECW1	370.967	997.828	1.43	0.000891251	up
SIX5	110.639	236.257	1.09	0.000933254	up
CUL4B	349.273	881.436	1.34	0.000933254	up
LDHA	673.286	102.77	-2.71	0.000947327	down
SULT1A3	48.811	187.615	1.94	0.000954993	up
CHKK	334.088	3588.299	3.43	0.001059254	up
ATP6V1A	178.975	384.84	1.1	0.001071519	up
SLC33A1	2034.517	9413.207	2.21	0.00108893	up
GHR	172.467	1856.252	3.43	0.00109144	up
ACAD10	154.027	354.229	1.2	0.001096478	up
MIS18BP1	84.607	264.607	1.65	0.001096478	up
ZMAT3	126.91	269.13	1.08	0.001122018	up
SIAH1	83.522	206.012	1.3	0.001122018	up
ERG	101.962	712.076	2.8	0.001174898	up
KRT20	374.374	139.647	-1.42	0.001183314	down
RPL8	1789.225	320.22	-2.48	0.00119234	down
CYCS	889.633	110.329	-3.01	0.001196465	down
TIAM1	273.344	889.638	1.7	0.001258925	up
B4GALT1	68.336	205.013	1.59	0.001258925	up
TGFBI	566.901	3119.184	2.46	0.0013122	up
FBXO44	86.776	239.972	1.47	0.001318257	up
CHCHD2	2378.631	538.281	-2.14	0.001333214	down
RHOA	2374.381	1067.955	-1.15	0.001376892	down
SLC30A9	55.32	171.817	1.64	0.001380384	up
SRCIN1	400.254	973.658	1.28	0.001412538	up
BCL2L13	44.473	156.212	1.81	0.001479108	up
SMARCA1	99.792	268.421	1.43	0.001548817	up
CAD	590.872	1809.909	1.62	0.001548817	up
ORC5	41.219	148.852	1.85	0.001584893	up
H3F3A	702.071	1718.546	1.29	0.001584893	up
MXD1	44.473	120.456	1.44	0.001584893	up
HBB	834.728	318.856	-1.39	0.001586719	down
RPL29	1992.276	713.565	-1.48	0.001607681	down
LDHB	456.724	65.176	-2.81	0.001608792	down
RELB	79.183	371.474	2.23	0.001659587	up
PLCB1	80.268	483.293	2.59	0.001686553	up
CCKBR	1279.437	6128.394	2.26	0.001698244	up
USP33	65.082	143.578	1.14	0.001737801	up
CASP7	632.915	2128.864	1.75	0.001778279	up
CTSS	1876.276	5572.297	1.57	0.001778279	up
RPS3	1274.527	610.119	-1.06	0.001815098	down
ACBD5	3006.788	8572.542	1.51	0.001819701	up

CD63	3646.381	968.394	-1.91	0.00185012	down
LSM14A	37.964	675.85	4.15	0.001862087	up
IL1B	424.34	7799.019	4.2	0.001905461	up
MAP3K9	35.795	81.679	1.19	0.001949845	up
IGJ	1993.347	315.081	-2.66	0.00195254	down
LTF	318.317	149.109	-1.09	0.002088334	down
RPL34	2558.201	1247.491	-1.04	0.002131081	down
ALDH1A3	366.167	1277.279	1.8	0.002137962	up
HLA-DRA	3846.368	1424.936	-1.43	0.002174203	down
EEF1B2	1902.872	251.299	-2.92	0.002178713	down
COL2A1	244.948	563.328	1.2	0.002187762	up
SP140	194.831	1476.616	2.92	0.002187762	up
HSPA8	973.642	318.566	-1.61	0.002250609	down
APBB2	1213.016	10604.455	3.13	0.002290868	up
IER2	3738.273	456.843	-3.03	0.002318996	down
SSR4	3216.263	1274.283	-1.34	0.002320064	down
CARD6	35.795	130.164	1.86	0.002344229	up
PABPC1	4208.668	789.802	-2.41	0.00245584	down
IFITM3	625.723	171.157	-1.87	0.002491724	down
CLDN4	360.402	757.952	1.07	0.002511886	up
RPL4	4675.725	1100.981	-2.09	0.00268349	down
TSPAN8	1284.896	244.898	-2.39	0.002791901	down
CSF3	88.945	331.403	1.9	0.003090295	up
DAPK1	1019.28	2410.029	1.24	0.003162278	up
DDX58	63.997	183.856	1.52	0.003162278	up
DHFR	46.642	114.171	1.29	0.003235937	up
SYNPO2L	2658.175	835.282	-1.67	0.00325237	down
RPL27	882.103	408.503	-1.11	0.003257617	down
EGR1	515.232	1567.314	1.61	0.003311311	up
FGF2	2602.302	10591.158	2.03	0.003311311	up
HCAR2	755.12	2689.388	1.83	0.003388442	up
HERC5	3122.457	15270.567	2.29	0.003388442	up
HES1	1882.24	8499.928	2.18	0.003467369	up
RPS19	1183.634	392.734	-1.59	0.003501064	down
HMOX1	2336.88	6199.207	1.41	0.003548134	up
IFIT1	162.144	602.047	1.89	0.003548134	up
IFIT2	914.105	3824.926	2.07	0.003548134	up
IFIT3	616.332	1820.43	1.56	0.003630781	up
KIF5B	61.828	150.299	1.28	0.003715352	up
AKR1B10	4621.961	1761.379	-1.39	0.003742829	down
IL24	10698.585	29947.177	1.49	0.003801894	up
IL2RG	59.262	251.434	2.09	0.003801894	up
ISG15	2345.01	9076.236	1.95	0.003890451	up
ISG20	31.456	311.538	3.31	0.003890451	up

RPL21	823.433	353.829	-1.22	0.003906609	down
MAPK13	121.443	453.713	1.9	0.003981072	up
ATP4A	1089.662	480.891	-1.18	0.004038313	down
MUC2	43.308	101.692	1.23	0.004064433	up
MYH15	507.991	1534.613	1.6	0.004073803	up
NFKBIA	4086.361	8804.961	1.11	0.004120975	up
NR4A3	32.069	137.96	2.11	0.004130475	up
NUDC	2514.025	7972.317	1.67	0.004168694	up
RPS29	2301.826	564.904	-2.03	0.004168694	down
SLC25A5	580.921	201.523	-1.53	0.004280554	down
RPS20	992.188	311.971	-1.67	0.0043722	down
RPSA	2672.447	1006.929	-1.41	0.004377236	down
FTL	572.861	147.201	-1.96	0.004559319	down
TFF1	214.667	30.8	-2.8	0.004571935	down
RPL23A	97.228	19.225	-2.34	0.004633402	down
RPLP0	2342.116	536.932	-2.13	0.004664445	down
OAS1	188.738	475.315	1.33	0.004677351	up
OASL	180.139	431.874	1.26	0.004897788	up
PTGS1	4913.224	10332.876	1.07	0.005011872	up
RPL41	104.381	16.171	-2.69	0.005109754	down
S100A9	145.157	335.221	1.21	0.005128614	up
CA2	1887.114	623.638	-1.6	0.005191585	down
SERPINE1	92.241	328.52	1.83	0.005495409	up
HLA-C	1620.018	475.399	-1.77	0.005530953	down
SLC4A11	68.336	224.345	1.72	0.005623413	up
TICAM1	288.346	801.555	1.48	0.005623413	up
TNFSF15	8850.26	20297.355	1.2	0.005754399	up
ANXA10	932.751	455.987	-1.03	0.005786287	down
TP53INP1	1203.156	3685.406	1.62	0.006025596	up
ZFP36	4073.208	8507.089	1.06	0.006025596	up
LIPF	862.214	114.571	-2.91	0.006046444	down
MT1H	2321.825	990.796	-1.23	0.006072954	down
ZFP36L2	2704.281	9919.346	1.88	0.006309573	up
GOSR1	173.907	507.468	1.55	0.006309573	up
RPS15A	3652.182	1736.592	-1.07	0.006496808	down
PGC	348.276	41.712	-3.06	0.006558432	down
INPP5F	21.035	67.169	1.68	0.006606934	up
CHMP2B	208.376	564.389	1.44	0.006606934	up
CDCA7L	1137.457	2805.605	1.3	0.00676083	up
CHAD	1847.913	8230.025	2.16	0.007014553	up
RPS27	433.482	149.161	-1.54	0.007021017	down
NPEPL1	10941.257	34696.22	1.67	0.007079458	up
S100P	678.724	195.697	-1.79	0.007207755	down
MT1X	1785.241	837.185	-1.09	0.007282827	down

EEF1A1	369.278	114.441	-1.69	0.007411396	down
CORO7	2061.291	4694.099	1.19	0.007413102	up
CLEC2B	3912.082	9716.499	1.31	0.007585776	up
TUBA1C	224.884	91.173	-1.3	0.007633083	down
TRAPPC2	82.179	171.635	1.06	0.007762471	up
RPS18	3452.189	1137.93	-1.6	0.007956096	down
RPS4X	1642.861	621.752	-1.4	0.007965261	down
GKN1	1378.328	228.667	-2.59	0.008248975	down
CRNKL1	300.132	817.148	1.45	0.008317638	up
DDX5	6427.4571	21247.896	1.73	0.00851138	up
RPS12	778.126	282.726	-1.46	0.008582227	down
NR2E1	699.031	2506.942	1.84	0.008609938	up
RAG2	86.358	226.718	1.39	0.008669619	up
GPC1	24.671	76.096	1.63	0.008689604	up
NPY6R	349.3	795.558	1.19	0.008709636	up
CTSE	267.778	88.739	-1.59	0.008763952	down
PACSIN3	690.265	1505.479	1.13	0.008912509	up
